# A Cell Biologist’s Field Guide to Aurora Kinase Inhibitors

**DOI:** 10.3389/fonc.2015.00285

**Published:** 2015-12-21

**Authors:** Christian O. de Groot, Judy E. Hsia, John V. Anzola, Amir Motamedi, Michelle Yoon, Yao Liang Wong, David Jenkins, Hyun J. Lee, Mallory B. Martinez, Robert L. Davis, Timothy C. Gahman, Arshad Desai, Andrew K. Shiau

**Affiliations:** ^1^Small Molecule Discovery Program, Ludwig Institute for Cancer Research, La Jolla, CA, USA; ^2^Laboratory of Chromosome Biology, Ludwig Institute for Cancer Research, La Jolla, CA, USA; ^3^Department of Cellular and Molecular Medicine, University of California San Diego, La Jolla, CA, USA

**Keywords:** Aurora kinase inhibitors, AZD1152, ZM447439, Hesperadin, MLN8237, MLN8054, MK-5108, MK-8745

## Abstract

Aurora kinases are essential for cell division and are frequently misregulated in human cancers. Based on their potential as cancer therapeutics, a plethora of small molecule Aurora kinase inhibitors have been developed, with a subset having been adopted as tools in cell biology. Here, we fill a gap in the characterization of Aurora kinase inhibitors by using biochemical and cell-based assays to systematically profile a panel of 10 commercially available compounds with reported selectivity for Aurora A (MLN8054, MLN8237, MK-5108, MK-8745, Genentech Aurora Inhibitor 1), Aurora B (Hesperadin, ZM447439, AZD1152-HQPA, GSK1070916), or Aurora A/B (VX-680). We quantify the *in vitro* effect of each inhibitor on the activity of Aurora A alone, as well as Aurora A and Aurora B bound to fragments of their activators, TPX2 and INCENP, respectively. We also report kinome profiling results for a subset of these compounds to highlight potential off-target effects. In a cellular context, we demonstrate that immunofluorescence-based detection of LATS2 and histone H3 phospho-epitopes provides a facile and reliable means to assess potency and specificity of Aurora A versus Aurora B inhibition, and that G2 duration measured in a live imaging assay is a specific readout of Aurora A activity. Our analysis also highlights variation between HeLa, U2OS, and hTERT-RPE1 cells that impacts selective Aurora A inhibition. For Aurora B, all four tested compounds exhibit excellent selectivity and do not significantly inhibit Aurora A at effective doses. For Aurora A, MK-5108 and MK-8745 are significantly more selective than the commonly used inhibitors MLN8054 and MLN8237. A crystal structure of an Aurora A/MK-5108 complex that we determined suggests the chemical basis for this higher specificity. Taken together, our quantitative biochemical and cell-based analyses indicate that AZD1152-HQPA and MK-8745 are the best current tools for selectively inhibiting Aurora B and Aurora A, respectively. However, MK-8745 is not nearly as ideal as AZD1152-HQPA in that it requires high concentrations to achieve full inhibition in a cellular context, indicating a need for more potent Aurora A-selective inhibitors. We conclude with a set of “good practice” guidelines for the use of Aurora inhibitors in cell biology experiments.

## Introduction

Aurora kinases were discovered in the mid-nineties in *Drosophila* and yeast (1, 2). Whereas yeasts only have one Aurora kinase, metazoans generally have two, named Aurora A and B. Mammals, but not other vertebrates, also have a third family member, Aurora C. Aurora A localizes to centrosomes and spindle microtubules and plays important roles in centrosome maturation, controlling spindle length and bipolarity, asymmetric cell division, and promoting mitotic entry both in unperturbed cells and following DNA damage (3, 4). Aurora B localizes to chromosomes/inner centromeres and the spindle midzone and is implicated in many processes including chromosome condensation, chromosome biorientation on the spindle, and cytokinesis (5–7). Aurora C is expressed in testis (8), where it exhibits tissue-specific functions (9, 10), and in oocytes, where it contributes to early embryonic divisions by providing functions associated with Aurora B in somatic cells (11–14). In addition, Aurora C is aberrantly expressed in cancer cells (15).

Due to their closely related kinase domains (72% identity for the human proteins), Aurora A and B exhibit similar protein substrate preferences *in vitro* (16–19). *In vivo*, their distinct substrate specificities, localization patterns, and functions arise from interactions with specialized binding partners (3, 4). Aurora B is largely found as part of the four-subunit chromosomal passenger complex (CPC) (5–7) whose three other members – INCENP, survivin, and borealin – localize the kinase to the centromere and the anaphase spindle. INCENP also activates Aurora B via a two-step mechanism (20–22). The IN box at the INCENP C-terminus first wraps around the N-terminal lobe of Aurora B, stimulating autophosphorylation of the activation loop residue Thr 232 (23). This event allows Aurora B to phosphorylate serines in the TSS motif adjacent to the IN box, which generates a feedforward loop by further augmenting INCENP’s ability to bind and activate Aurora B.

Aurora A has multiple regulators, with the best-studied one being TPX2, which activates the kinase and targets it to spindle microtubules (24–26). Structural studies have shown that the TPX2 N-terminus binds the N-terminal lobe of Aurora A, in a manner distinct from how the INCENP IN box binds Aurora B, facilitating the alignment of residues essential for substrate binding and catalysis (27–29). In biochemical assays, binding of the TPX2 N-terminus increases autophosphorylation of the activation loop residue Thr 288 (28, 30, 31). As in the case of Aurora B, phosphorylation of this threonine [which readily occurs *in vitro* even in the absence of TPX2 or other activators (16)] promotes high levels of kinase activity (16, 28, 32). However, recent studies have unexpectedly revealed that this autophosphorylation event is not essential for TPX2 stimulation of Aurora A kinase activity; fully dephosphorylated Aurora A bound to TPX2 exhibits robust enzymatic activity (28, 32). The relative contributions of TPX2 binding and Thr 288 phosphorylation to different cellular Aurora A functions is an active area of investigation.

Coincident with the delineation of their cellular roles, the Aurora kinases were also found to be amplified/overexpressed in cancer (33, 34). Functional studies of Aurora A revealed a potential role in tumor initiation and growth – increased expression of Aurora A transformed rodent fibroblasts (albeit weakly) and promoted their ability to form tumors *in vivo* (35, 36). In addition, elevated Aurora A activity was shown to confer resistance to taxol-mediated apoptosis in cancer cells (37). The Aurora kinases therefore emerged as attractive drug targets in cancer and became the focus of intense drug discovery efforts (38–41).

**Figure 1:**
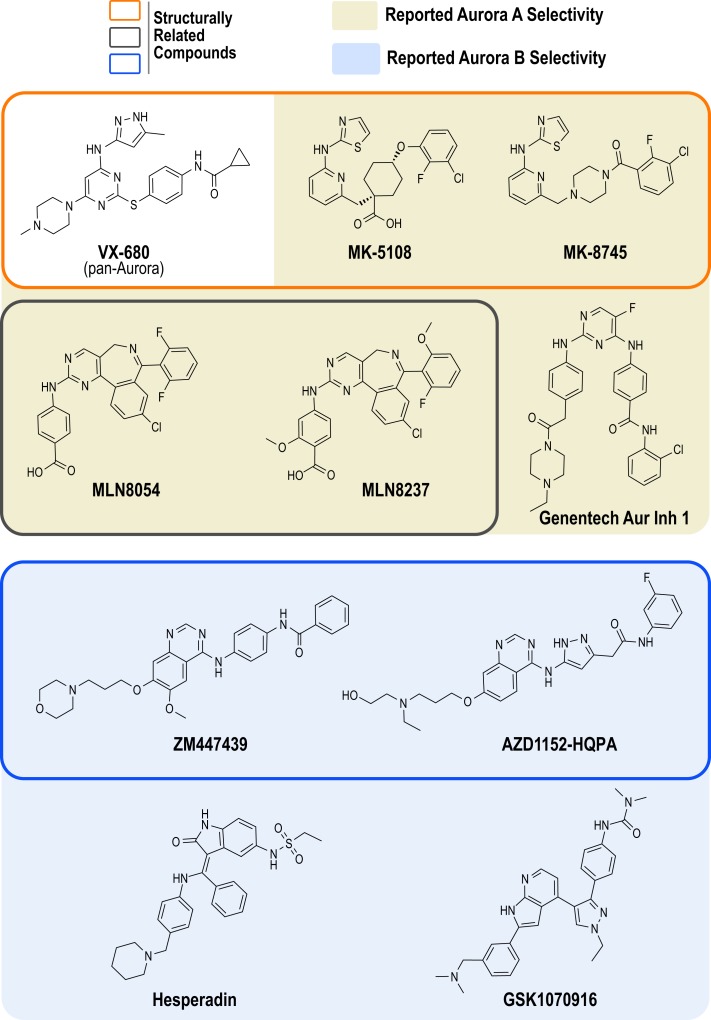
**Chemical structures of Aurora kinase inhibitors analyzed in this study**. The chemical structures of the 10 commercially available compounds characterized in this study are depicted (see Table S1 in Supplementary Material for suppliers). Outlined boxes group chemically related inhibitors: Pyrazolo-pyrimidine class (Vertex/Merck/Banyu) – MK-5108 and MK-8745 were derived from VX-680 (*orange outlined box*); Benzazepine class (Millenium/Takeda) – MLN8237 was derived from MLN8054 (*gray outlined box*); and Quinazoline class (Astra Zeneca) – AZD1152-HQPA was derived from ZM447439 (*blue outlined box*).

At least 30 Aurora kinase inhibitors have been evaluated preclinically or clinically as potential oncology therapeutics (38). The development of these inhibitors has typically involved high throughput biochemical assays using purified proteins, structure-based drug design, cellular biomarker assays (primarily Aurora A Thr 288 phosphorylation and Aurora B-mediated phosphorylation of its canonical substrate, histone H3), cellular proliferation/cytotoxicity assays, and xenograft models in mice (39). The products of the vast majority of these programs have been compounds that potently inhibit all three Aurora kinases (A, B and C), as best exemplified by the first clinically tested Aurora kinase inhibitor, the Vertex/Merck pyrazolo-pyrimidine compound VX-680 (MK-0457, tozasertib; Figure 1) (42, 43). However, compounds that exhibit preference for Aurora A or B/C have also been developed. In 2003, two pioneering academic-industrial collaborations described two distinct Aurora B inhibitors: the indolinone Hesperadin and the quinazoline ZM447439 [Figure 1; (44, 45)]. The latter compound was further optimized to produce the structurally related pro-drug AZD1152 (barasertib); barasertib is metabolized to the active form AZD1152-HQPA, which lacks the phosphate group present on AZD1152 and is the form typically used in biochemical and cell-based studies (Figure 1) (46, 47). In 2007, Millenium (now Takeda) described the first Aurora A-selective inhibitor, the benzazepine MLN8054 (48–51), which, due to central nervous system side effects (52, 53), was replaced as the lead clinical candidate by the derivative MLN8237 (alisertib; Figure 1) (49, 54, 55). In parallel, optimization of the VX-680 scaffold by Merck/Banyu/Vertex resulted in the Aurora A-selective inhibitors MK-5108 (VX-689) (56) and MK-8745 (57, 58) (Figure 1). More recently, other structurally unrelated Aurora A- and B-selective inhibitors have been described, such as the bisanilinopyrimidine inhibitor Genentech Aurora Inhibitor 1 (optimized to target Aurora A) (59) and the azaindole-based GSK1070916 (optimized to target Aurora B/C) (60–62) (Figure 1).

While these compounds were developed with a primary emphasis on therapeutic benefit, they were rapidly adopted by academic investigators as chemical tools for biochemical, structural, and cell biological studies (63). Application of these small molecules has complemented genetic knockdown and immunodepletion approaches because their inhibitory effects exhibit high penetrance/rapid onset and can be readily reversed. Their use has been wide ranging and influential, resulting in a large body of work defining Aurora kinase cellular functions, identifying potential substrates, and elucidating molecular mechanisms of kinase activation (63).

Despite the common use of several Aurora inhibitors by the cell biology community, a systematic comparison of these compounds in quantitative *in vitro* and cellular assays has been lacking. Further, it is presently unclear how the potencies, selectivities, off-target profiles, and cellular efficacies of the most frequently used inhibitors compare to those of more recently described, potentially improved molecules. Here, we fill this gap by profiling the 10 commercially available inhibitors shown in Figure 1 in biochemical and cell-based assays. Our results highlight significant challenges in the selective inhibition of Aurora A, identify the best compounds for specific and potent targeting of Aurora A and Aurora B, and lead us to present a set of recommendations for the experimental use of these compounds.

## Results

### Quantitative Biochemical Analysis of Inhibitor Potency and Specificity

**Figure 2:**
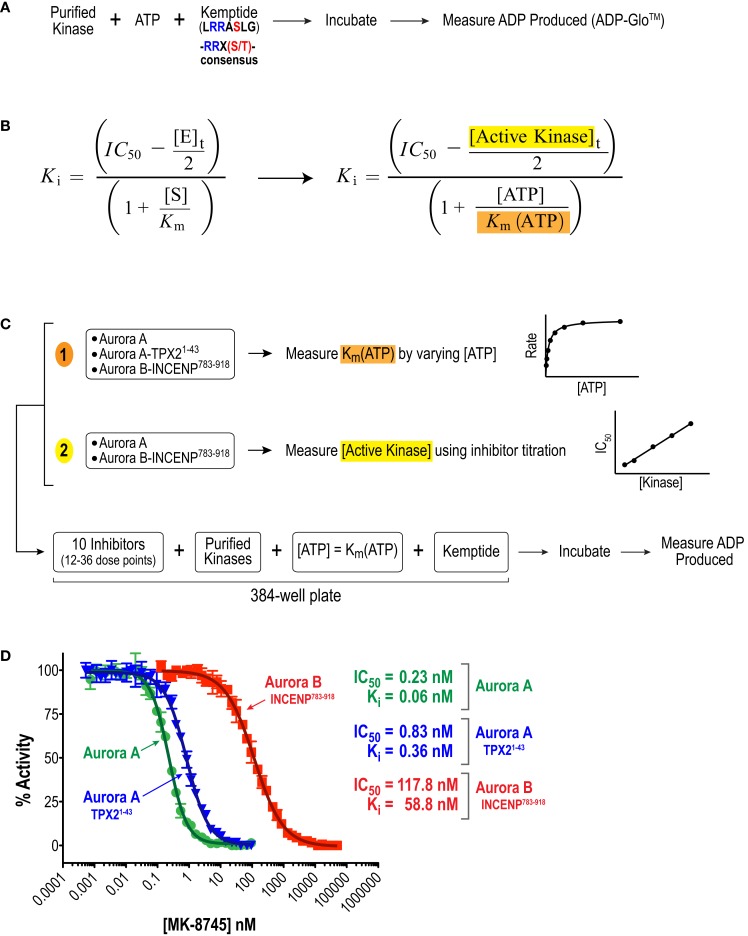
**Approach used to quantify inhibitor potency and selectivity *in vitro***. **(A)** Schematic of protocol used to measure kinase activity by quantifying ADP production using the luminescence-based assay technology, ADP-Glo™ (Promega). **(B)** Equation used to convert measured IC_50_ values to *K*_i_ values. This equation simplifies to the more familiar Cheng and Prusoff approximation in cases where *K*_i_ >> [*E*]_t_ (65). **(C)** Schematic of approach used to quantify inhibitor potencies. First, key variables, (1) *K*_m_(ATP) and (2) [*E*]_t_ = [Active kinase]_t_, required for the IC_50_ conversion were measured. Second, a multi-point inhibitor dose titration was performed in 384-well plates under conditions where [ATP] = *K*_m_(ATP) to determine IC_50_ values. **(D)** Example datasets for MK-8745 showing the measured IC_50_s and the *K*_i_s calculated from them using the equation in **(B)**.

We began by analyzing the inhibitory properties of the 10 compounds in Figure 1 (see Table S1 in Supplementary Material for suppliers) on the *in vitro* activities of full-length human Aurora A, alone or bound to an activating N-terminal peptide fragment of TPX2 (residues 1–43), and full-length human Aurora B bound to a C-terminal fragment of INCENP (residues 783–918, which includes both the IN box and TSS motifs; Figure 2A). As the large number of required measurements (3–11 independent sets of triplicate 12–36 point dose–response curves per compound) precluded the use of conventional radiometric substrate phosphorylation assays, we explored several recently developed high throughput methods for measuring kinase catalytic activity in multi-well microplate format (64). To allow comparison of Aurora A and B activity under similar reaction conditions (Figure 2A), we ultimately selected a sensitive assay format that monitors ADP production (ADP-Glo™ – see Materials and Methods). Briefly, kinase reactions (with or without inhibitors) were performed in 384-well plates with saturating amounts of a generic peptide substrate containing the Aurora kinase consensus phosphorylation motif (Kemptide – L*RR*A*S*LG; Aurora kinase consensus *RR*X*S*/*T*). After a defined incubation period, an enzyme cocktail was added to terminate the reaction and convert any remaining ATP to cyclic AMP. This was followed by a second enzyme cocktail that converted the ADP produced by the kinase reaction to ATP and, in turn, the newly generated ATP to a luminescent signal via luciferase. The resulting luminescence was then quantified using a microplate reader (Figure 2A).

Given the high affinities of the inhibitors and the enzyme concentrations required for sufficient signal-to-noise, some experiments were performed under “tight binding” conditions (66). Under these conditions, the total active enzyme concentration ([*E*]_t_) matches or exceeds the dissociation constant for the enzyme/inhibitor complex (*K*_i_); therefore, the assumption that the concentration of free inhibitor is equivalent to that added to the reaction is not valid. The *K*_i_ was therefore calculated from the measured IC_50_ (concentration for half maximal inhibition) using the equation shown in Figure 2B (66–69). Use of this equation assumes that the compounds act through a direct competitive mechanism and requires that the substrate concentration [S], which in this case is [ATP], *K*_m_(ATP), and [*E*]_t_ be precisely known. Therefore, for all three enzyme species employed in this analysis, we first measured *K*_m_(ATP) through an ATP titration (Figure 2C), and then performed all reactions at [ATP] = *K*_m_(ATP) so that the denominator simplified to two. We also measured [*E*]_t_ using inhibitor titrations under conditions where [*E*]_t_ and [I] >> *K*_i_, which enables the approximation that IC_50_ ~ [*E*]_t_/2 (Figure 2C). Example dose–response curves for MK-8745, the measured IC_50_s, and the resulting *K*_i_ values are depicted in Figure 2D. *K*_i_ values for all 10 inhibitors for Aurora A, Aurora A/TPX2^1–43^ and Aurora B/INCENP^783–918^ are reported in Table 1. The *K*_i_ values were used to calculate the selectivity ratios of each inhibitor for the three enzyme species (Table 2). Because of the extremely slow on-rate of GSK1070916 for Aurora B/INCENP, this *K*_i_ could not be accurately measured under our conditions, so the previously described value (61) was used for selectivity analysis. For reference, the published Aurora A/TPX2^1–43^*K*_i_ for GSK1070916 is also presented in Table 1.

**Table 1:** **Biochemical inhibition constants of the Aurora inhibitor panel**.

	Aurora A	Aurora A-TPX2^1–43^	Aurora B-INCENP^783–918^
	
	*K*_i_ Mean ± SD (nM)	*K*_i_ Mean ± SD (nM)	*K*_i_ Mean ± SD (nM)
VX-680	1.03 ± 0.18 (*n*=11)	4.55 ± 0.57 (*n*=9)	1.11 ± 0.20 (*n* = 9)

MK-5108	<0.01^a^ (*n* = 4)	0.04 ± 0.008 (*n* = 3)	1.49 ± 0.21 (*n* = 3)
MK-8745	0.06 ± 0.004 (*n* =4)	0.41 ± 0.06 (*n* =3)	66.8 ± 19.6 (*n* =3)
MLN8054	0.15 ± 0.01 (*n* =4)	0.80 ± 0.09 (n = 3)	1.65 ± 0.36 (n = 3)
MLN8237	0.04 ± 0.007 (*n* =3)	0.23 ± 0.02 (*n* = 3)	1.10 ± 0.23 (*n* = 3)
Genentech Aurora Inhibitor 1	0.57 ± 0.06 (*n* = 3)	0.24 ± 0.02 (*n* = 3)	156.2 ± 33.7 (*n* = 3)

ZM447439	55.5 ± 8.2 (*n* = 4)	336.8 ± 50.5 (*n* = 3)	1.83 ± 0.28 (*n* = 3)
AZD1152-HQPA	83.8 ± 14.2 (*n* = 4)	351.9 ± 64.1 (*n*=3)	0.02 ± 0.009 (n = 3)
Hesperadin	1.21 ± 0.14 (n = 3)	1.37 ± 0.12 (*n* = 3)	0.03 ± 0.014 (*n* = 3)
GSK1070916	16.1 ± 1.3 (*n* =3)	130.2 ± 33.2 (*n* =3); 490^b^	0.38^b^

*^a^Upper bound*.

*^b^Values from Ref. (61)*.

**Table 2:** ***In vitro* selectivity ratios of the Aurora inhibitor panel (fold difference in potency calculated by dividing *K*_i_ values measured for each kinase)**.

	Aur A versus Aur A-TPX2^1–43^	Aur A versus Aur B-INCENP^783–918^	Aur A-TPX2^1–43^ versus Aur B-INCENP^783–918^	Aur B-INCENP^783–918^ versus Aur A	Aur B-INCENP^783–918^ versus Aur A-TPX2^1–43^
VX-680	4.4	1.1	0.2	0.9	4.1
MK-5108	>4	>149	39.1		
MK-8745	6.3	1030	162		
MLN8054	5.5	11.3	2.1		
MLN8237	5.5	26.8	4.9		
Genentech Aurora Inhibitor 1	0.4	274	654		
ZM447439	6.1			30	184
AZD1152-HQPA	4.2			3759	15779
Hesperadin	1.1			40.2	45.3
GSK1070916	8.1			42.2	343

Consistent with previously reported measurements [Table S2 in Supplementary Material; (42, 70)], the well-characterized pan-Aurora inhibitor VX-680 inhibited both Aurora A and Aurora B/INCENP^783–918^ with essentially identical potencies [(*K*_i_=1.0 nM); Table 1]. This compound was therefore included as a reference in the assays for the remaining nine compounds. We note that, based on significant differences in enzyme construct design, sources, purification methods, as well as assay conditions/readouts, it is not straightforward to compare our *K*_i_ values to values in the literature (which are, in many cases, wide ranging). Therefore, for all compounds (beyond VX-680), we largely restrict our discussion of prior work to trends in potency and selectivity ratios.

As expected, all of the compounds reported to be Aurora B-selective were extremely potent Aurora B/INCENP^783–918^ inhibitors with a rank order of potency of AZD1152-HQPA > Hesperadin >> GSK1070916 (61) > ZM447439 (Table 1) and exhibited a high selectivity (minimum of 30-fold) for Aurora B/INCENP^783–918^ over Aurora A (Table 2). Although our mean Aurora B/INCENP^783–918^*K*_i_ value (0.02 nM) (Table 1) for AZD1152-HQPA is ~18-fold lower than that previously reported [0.36 nM; Table S2 in Supplementary Material; (46, 47)], this is also the case for the Aurora A *K*_i_ values [~16-fold; 84 nM in this study (Table 1) versus 1.4 µM from published work (Table S2 in Supplementary Material; (46, 47))]. Thus, the selectivity ratio calculated from our measurements is similar to that which can be derived from prior work (3760-fold versus 3890-fold) (Table 2 and Table S2 in Supplementary Material).

All of the described Aurora A-selective inhibitors had subnanomolar *K*_i_s for Aurora A, with a rank order of potency of MK-5108 > MLN8237 > MK-8745 > MLN8054 > Genentech Aurora Inhibitor I (Table 1). MK-5108 exhibited an inhibition constant below what we could accurately measure (≤10 pM). All of these compounds inhibited Aurora B/INCENP^783–918^ less potently than Aurora A, with MK-8745 exhibiting the highest selectivity for Aurora A (1,030-fold) and MLN8054 and MLN8237 the lowest (11- and 27-fold, respectively) (Table 2). The selectivity measured for MLN8054 was lower than the published value [Table S2 in Supplementary Material; 43-fold (48)], possibly in part because this previous calculation was based on IC_50_s, which can be highly dependent upon [ATP], *K*_m_(ATP), and potentially [*E*]_t_ (Figure 2B). In agreement with this, the *K*_i_-based selectivity ratio we report for MLN8054 (11-fold) (Table 2) is close to that described in a structural, biochemical, and mutational analysis of the Aurora A inhibitory properties of MLN8054 (6-fold) (71).

Given the importance of TPX2 as an Aurora A regulator, we also assessed the inhibitory activity of all 10 compounds on the Aurora A/TPX2^1–43^ complex. Excluding Genentech Aurora Inhibitor I and Hesperadin, the presence of TPX2^1–43^ weakened binding by 4- to 8.1-fold (Tables 1 and 2). Intriguingly, TPX2^1–43^ increased the affinity of Genentech Aurora Inhibitor I for Aurora A 2.5-fold, whereas Hesperadin binding was unaffected (Tables 1 and 2). Decreased Aurora A *K*_i_s in the presence of TPX2^1–43^ has been previously reported for VX-680, MK-5108, MLN8054, and MLN8237 [Table S2 in Supplementary Material; (70–72)].

Binding of the TPX2 N-terminus to Aurora A stabilizes a productive conformation of its substrate binding and catalytic elements [including the catalytic lysine (Lys 162), the αC helix which bears the glutamic acid (Glu 181) that interacts with Lys 162, the DFG motif, and the activation loop containing Thr 288] (20, 28). In contrast, inhibitors, such as VX-680, MLN8054, and quinazoline-class compounds, favor distorted inactive conformations of some or all of these elements (59, 71, 73–76). As suggested previously for VX-680 and a quinazoline resembling ZM447439 and AZD1152-HQPA (70), these opposing structural effects likely result in the decreased affinities of the majority of the inhibitors we characterized for the Aurora A/TPX2^1–43^ complex (Table 2). Conversely, based on their respective positions in the Aurora A and Aurora B binding pockets, Genentech Aurora Inhibitor I (59) and Hesperadin (20) are predicted to make minimal contact with the active site elements that move upon TPX2^1–43^ binding. This potentially explains the subtle changes in Aurora A *K*_i_s for these two compounds in the presence of TPX2^1–43^ (Table 2).

From a biochemical selectivity perspective, the *K*_i_ shifts driven by TPX2^1–43^ binding have important but different consequences for the Aurora A- and Aurora B-selective compounds. The selectivity ratios of GSK1070916, ZM447439, and AZD1152-HQPA (preference for Aurora B over A) increase to ≥184 in the presence of TPX2^1–43^ (Table 2). Conversely, the selectivity ratios of the Aurora A-selective inhibitors diminish significantly, with MLN8054 and MLN8237 exhibiting only two- and fivefold preference, respectively, for Aurora A/TPX2^1–43^ over Aurora B/INCENP^783–918^ (Table 2). Given the prevalent use of MLN8054 and MLN8237 as Aurora A-selective tools, these findings motivated us to analyze our inhibitor panel in a battery of cellular assays.

### Substrate Phosphorylation-Based Profiling of Aurora Inhibitors in HeLa Cells

The critical parameters influencing inhibitor choice for cell biologists are efficacy and specificity in a cellular context. Thus, we next focused on identifying robust and reproducible cellular readouts for Aurora A and Aurora B kinase activity and employed them to systematically profile inhibitors in dose–response in three cell lines commonly used in cell biological studies: HeLa cervical carcinoma, hTERT-RPE1 retinal pigment epithelial (hereafter referred to as RPE1), and U2OS osteosarcoma cells. Based on previous biochemical studies, it is known that many of the inhibitors we tested can inhibit Aurora C. However, based on our qPCR analysis and previously published work (15), Aurora C mRNA is expressed at low levels in HeLa and RPE1 cells, and only present at ~20% of Aurora B mRNA levels in U2OS cells (Figure S1A in Supplementary Material). Thus, we believe that the biological effects we detect are predominantly, if not exclusively, mediated by Aurora A and B.

**Figure 3:**
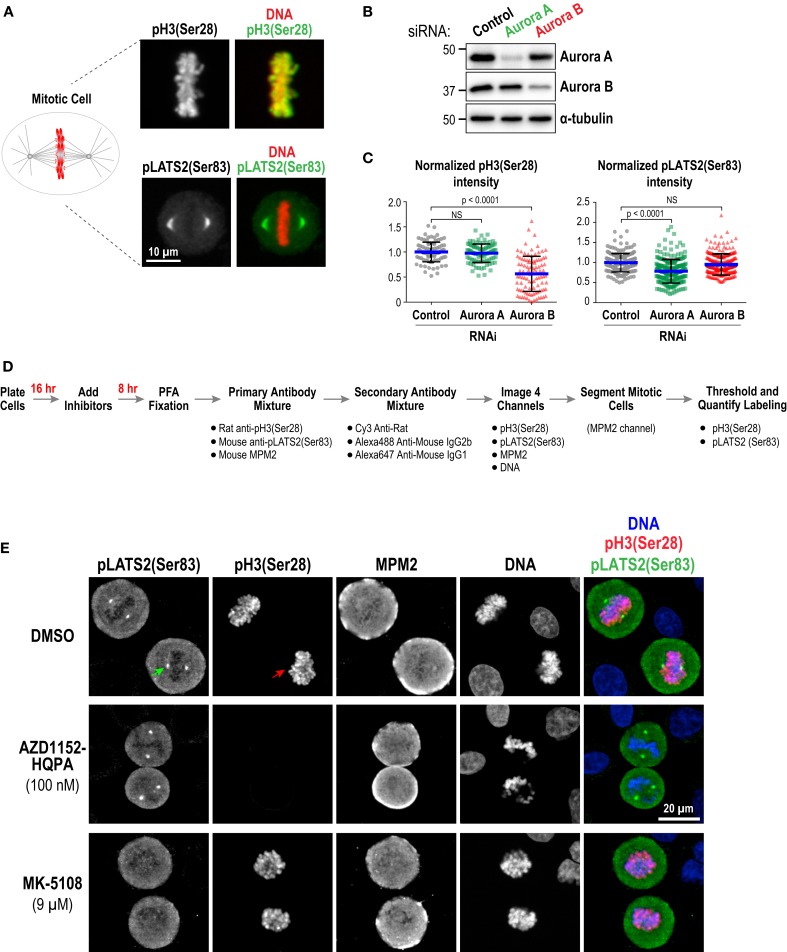
**Substrate phosphorylation-based analysis of Aurora inhibitor specificity and potency in a cellular context: assay validation and example images**. **(A)** Images of mitotic cells labeled for pH3(Ser 28) (*top panels*) and pLATS2(Ser 83) (*bottom panels*). Merges with DNA (*red*) are shown on the right. **(B)** Immunoblots of HeLa cells following Aurora A or Aurora B RNAi. α-tubulin serves as a loading control. Expression levels of Aurora A and B are reduced by ~90 and ~65%, respectively, under these conditions. **(C)** Quantification of pH3(Ser 28) (*left graph*) and pLATS2(Ser 83) (*right graph*) labeling for the indicated conditions. Blue lines indicate the mean; black error bars are the SD. *p*-values are from unpaired *t*-tests. **(D)** Schematic of procedure used to process cells for labeling and quantification of pH3(Ser 28) and pLATS2(Ser 83). **(E)** Example images of control DMSO-treated (*top row*) and inhibitor-treated (*bottom two rows*) cells. Arrows in the control DMSO-treated panels highlight pLATS2(Ser 83) labeling of spindle poles (*green arrow*) and pH3(Ser 28) labeling of mitotic chromatin (*red arrow*). The concentrations shown for AZD1152-HQPA and MK-5108 exemplify selective inhibition of Aurora B and Aurora A activity, respectively.

As a first approach, we performed immunofluorescence in fixed HeLa cells to detect phospho-epitopes associated with the activity of each kinase. Aurora A has multiple known substrates enriched at centrosomes/mitotic spindles, including the Hippo pathway kinase LATS2 (Ser 83) (77), TACC3 (Ser 558) (78–82), and Aurora A itself (Thr 288) (16, 17). We chose pLATS2(Ser 83) as a cellular readout for Aurora A activity because pilot experiments, guided by a prior study (83), indicated that robust, specific labeling could be obtained using a commercial monoclonal antibody (Clone ST-3B11) targeting this epitope (Figure 3A). Aurora B phosphorylates Ser 10 and Ser 28 in the N-terminal tail of histone H3 (84, 85) and reliable antibodies are commercially available for detecting these phospho-epitopes in cells (Figure 3A; Table S3 in Supplementary Material). We chose pH3(Ser 28) as the model substrate site because robust labeling could be achieved under fixation conditions compatible with pLATS2(Ser 83) labeling, allowing us to monitor activities of both Aurora A and B in the same cells in 96-well plates. We used RNAi (Figure 3B) to confirm that pH3(Ser 28) is sensitive to knockdown of Aurora B but not Aurora A, and that pLATS2(Ser 83) is significantly reduced by knockdown of Aurora A but not Aurora B (Figures 3B,C); the partiality of the RNAi likely accounts for the less-than-complete elimination of pLATS2 signal. pH3(Ser 10) behaved similarly to pH3(Ser 28) (Figure S1B in Supplementary Material), as expected (84, 85).

We employed the protocol described in Figure 3D to analyze substrate phosphorylation in HeLa cells following treatment with all 10 inhibitors in dose–response. Asynchronous cells were incubated with vehicle (DMSO) or different inhibitor doses for 8 h and then fixed and labeled with a mixture of three antibodies directed against pLATS2(Ser 83), pH3(Ser 28), and MPM2 [which detects mitotic phosphoepitopes; (86)]. While both the anti-pLATS2(Ser 83) and MPM2 antibodies are mouse monoclonals, they are of different IgG subclasses [IgG2b for anti-pLATS2(Ser 83) and IgG1 for MPM2], and can thus be detected with subclass-specific secondary antibodies (Table S3 in Supplementary Material).

**Figure 4:**
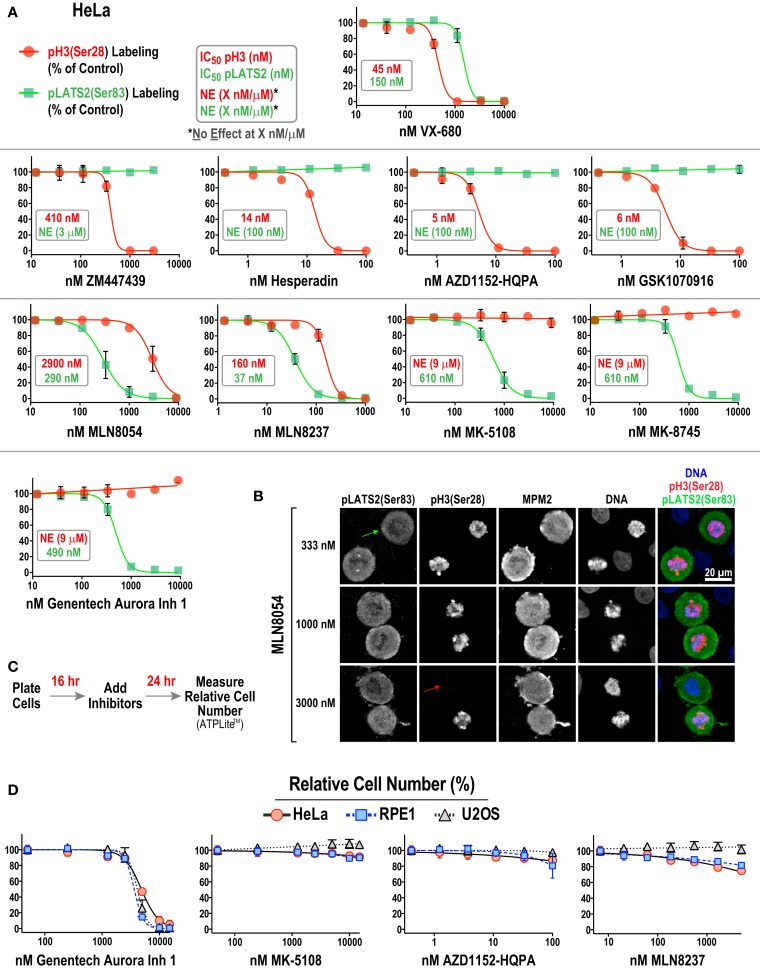
**Substrate phosphorylation-based analysis of all 10 Aurora inhibitors in HeLa cells**. **(A)** Dose–response curves measuring pH3(Ser 28) (*red circles and line*) and pLATS2(Ser 83) (*green squares and line*) labeling intensity for all 10 inhibitors in HeLa cells. Each point on the graphs represents the mean of measurements performed on four separate plates (average of 350 cells per point), normalized relative to control; the SD is plotted when larger than the symbol size. IC_50_ values are listed on graphs, when applicable, and were derived by computing dose–response curves using a 4-parameter, variable slope fit in GraphPad Prism. When there was no effect on labeling intensity at the highest tested concentration (X μM/nM), NE(X μM/nM) is indicated on the graphs, e.g., in AZD1152-HQPA graph (NE, 100 nM, *green text*) indicates no effect on pLATS2(Ser 83) labeling of 100 nM AZD1152-HQPA. **(B)** Example images of HeLa cells treated with MLN8054 at different doses highlighting partial (333 nM), selective (1000 nM) and non-selective (3000 nM) inhibition of pLATS2(Ser 83) labeling. Green and red arrows highlight cells lacking pLATS2(Ser 83) and pH3(Ser 28) labeling, respectively. **(C)** Protocol used to measure cellular proliferation after short-term (24 h) inhibitor exposure. Relative cell number was quantified by measuring ATP levels, using a luminescence-based assay (ATPLite™ from PerkinElmer). **(D)** Dose–response curves measuring cellular proliferation for the indicated four inhibitors in three cell lines: HeLa, RPE1, and U2OS. Each point represents the mean of six measurements from two independent experiments. Error bars are the SD. See Figure S2B in Supplementary Material for the graphs for the other six inhibitors.

In control mitotic cells, pLATS2(Ser 83) is concentrated in foci around the spindle poles (Figure 3E; *top row – green arrow*) and pH3(Ser 28) is on the mitotic chromatin (Figure 3E; *top row – red arrow)*. Selective kinase inhibition should result in loss of one signal but not the other, as illustrated by the example images for specific conditions in Figure 3E (*middle and bottom rows*). Cells were imaged in 4 channels to visualize pLATS2(Ser 83), pH3(Ser 28), MPM2, and DNA (labeled with Hoechst) and mitotic cells were segmented based on their bright MPM2 labeling (Figure 3E). Intensity and area thresholds were set to select the pLATS2(Ser 83) foci and the pH3(Ser 28)-labeled chromatin in their respective channels in DMSO-treated control cells and the same thresholds were applied for inhibitor-treated cells. The mean fluorescence intensity per pixel was measured to assess the activities of the kinases targeting these two substrate phosphorylation sites. The results of this analysis for all 10 inhibitors in dose–response are shown in Figure 4A.

Two major conclusions emerging from this dataset are:

(1) All four Aurora B-selective inhibitors can be used to specifically and potently inhibit H3(Ser 28) phosphorylation in cells. Consistent with the behavior of these compounds in the enzymatic assays described above, AZD1152-HQPA, Hesperadin, and GSK1070916 are extremely potent, completely eliminating pH3(Ser 28) labeling without affecting pLATS2(Ser 83) labeling at <100 nM concentrations.
(2) The inhibitors designed to target Aurora A require significantly higher concentrations for efficacy and exhibit greater variability with respect to specificity. MK-5108 and MK-8745, two related compounds (Figure 1), achieve specific Aurora A inhibition, as demonstrated by loss of pLATS2(Ser 83) labeling without reduction of pH3(Ser 28) labeling. However, both compounds require high micromolar concentrations for full efficacy (Figure 4A). In contrast, and consistent with the biochemical data, the commonly used MLN8054 and MLN8237 compounds have narrower specificity windows (10- and 4-fold, respectively), which makes it difficult to fully inhibit Aurora A without affecting Aurora B (Figure 4A). This point is illustrated by example images of MLN8054-treated HeLa cells at three different concentrations (Figure 4B). With careful optimization, these inhibitors can be employed for selective Aurora A inhibition, especially if the experimental goal is partial Aurora A inhibition. However, based on this dataset, MK-5108 and MK-8745 would be preferred for selectively targeting Aurora A.

Although similar to MK-5108 and MK-8745 in terms of Aurora A specificity, Genentech Aurora Inhibitor 1 led to significantly reduced proliferation and apoptotic cell death in HeLa cells within 24 h of treatment (Figures 4C,D; Figure S2A in Supplementary Material). This toxicity, which was also observed in U2OS and RPE1 cells (Figure 4D), is most likely due to off-target effects, as it is not observed with MK-5108, MLN8237, or AZD1152-HQPA (Figure 4D; Figure S2B in Supplementary Material). Therefore, the narrow window between efficacy and cytotoxicity of Genentech Aurora Inhibitor 1 suggests that it should not be used in routine cell culture experiments for Aurora A inhibition.

### Analysis of Inhibitor Efficacy in RPE1 and U2OS Cells Highlights Variation in Potency and Specificity Across Cell Lines

**Figure 5:**
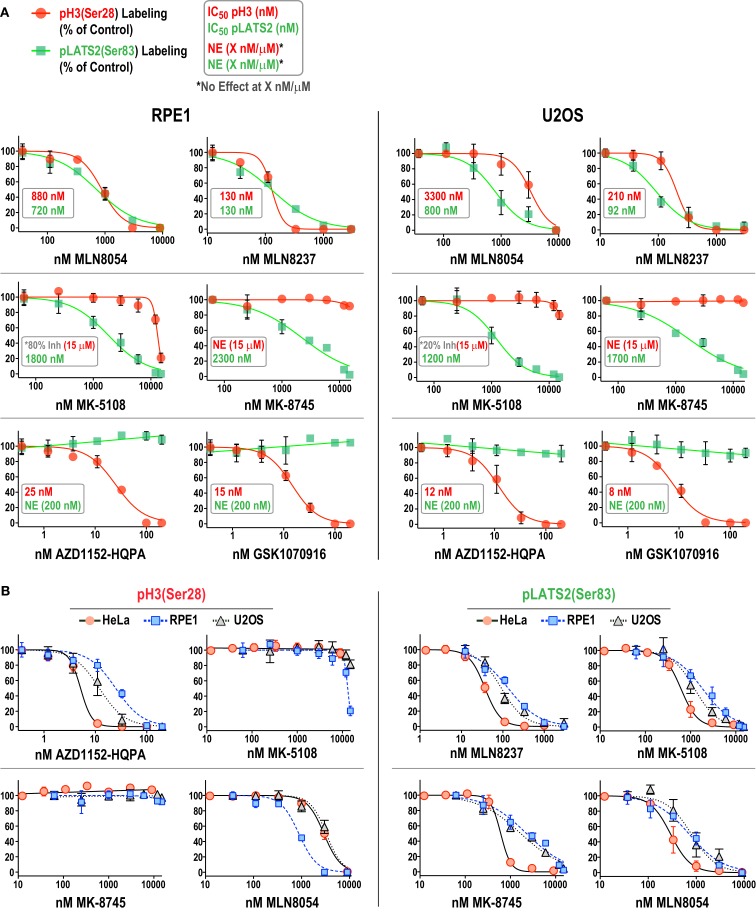
**Comparison of inhibitor specificity and potency between HeLa, RPE1, and U2OS cells**. **(A)** Dose–response curves measuring pH3(Ser 28) and pLATS2(Ser 83) labeling intensity for the six indicated inhibitors in RPE1 and U2OS cells, plotted and labeled as in Figure 4A. Note that for MK-5108 and pH3(Ser 28) labeling, where full inhibition was not achieved in the concentration range tested, the inhibition observed at the highest concentration tested is indicated on the graphs. Each point on the graphs represents the mean of measurements performed on four separate plates (average of 200 cells per point), normalized relative to control. **(B)** Dose–response data for pH3(Ser 28) (*left set of graphs*) and pLATS2(Ser 83) (*right set of graphs*) labeling intensity plotted for all three cell lines for the indicated inhibitors. Note that AZD1152-HQPA, which potently inhibits pH3(Ser 28) and is plotted on the left, has no effect on pLATS2(Ser 83) labeling in any cell line over the tested concentration range (0–200 nM), and is thus not plotted on the right; instead MLN8237 is plotted. See also Figure S2C in Supplementary Material.

We focused on additional characterization of the four inhibitors designed to target Aurora A that were not cytotoxic (MLN8054, MLN8237, MK-5108, MK-8745; Figure 4D; Figure S2B in Supplementary Material), and AZD1152-HQPA and GSK1070916, because they are chemically distinct (Figure 1) and the two most potent Aurora B inhibitors in the HeLa substrate phosphorylation assays (Figure 4A). As a first step, we analyzed substrate phosphorylation in RPE1 and U2OS cells for these six compounds (Figures 5A,B). This analysis revealed that the specificity window for certain inhibitors was significantly narrower in RPE1 and U2OS compared to HeLa cells, as best illustrated by MLN8054 and MLN8237 (compare Figure 5A with Figure 4A). In addition, inhibitor potency varied up to fourfold across the three cell lines (Figure 5B; Figure S2C in Supplementary Material). Regardless of the specific reasons for this variation (discussed below), our results underscore the technical importance of performing a dose–response analysis with the pLATS2(Ser 83) and pH3(Ser 28) labeling assay in all experimental cell lines in order to identify the minimum concentration required for selective and complete Aurora kinase inhibition. The results of this analysis confirm MK-5108 and MK-8745 as the current best Aurora A-specific inhibitors, with the latter exhibiting the least effect on pH3(Ser 28) at doses that eliminate pLATS2(Ser 83) labeling. We additionally note that H3(Ser 28) may be targeted by Aurora C in tissues/cell types where this kinase is expressed. As Aurora C mRNA is present at modest levels in U2OS cells and all pH3(Ser 28) signal is abolished by AZD1152-HQPA and GSK1070916 in this cell line, we believe any minor Aurora C activity that may be present is inhibited by these compounds, a conclusion that is consistent with published biochemical studies (46, 47, 61).

### Measurement of G2 Duration in a Live Imaging Assay Enables Assessment of Inhibitor Potency and Specificity for Aurora A

**Figure 6:**
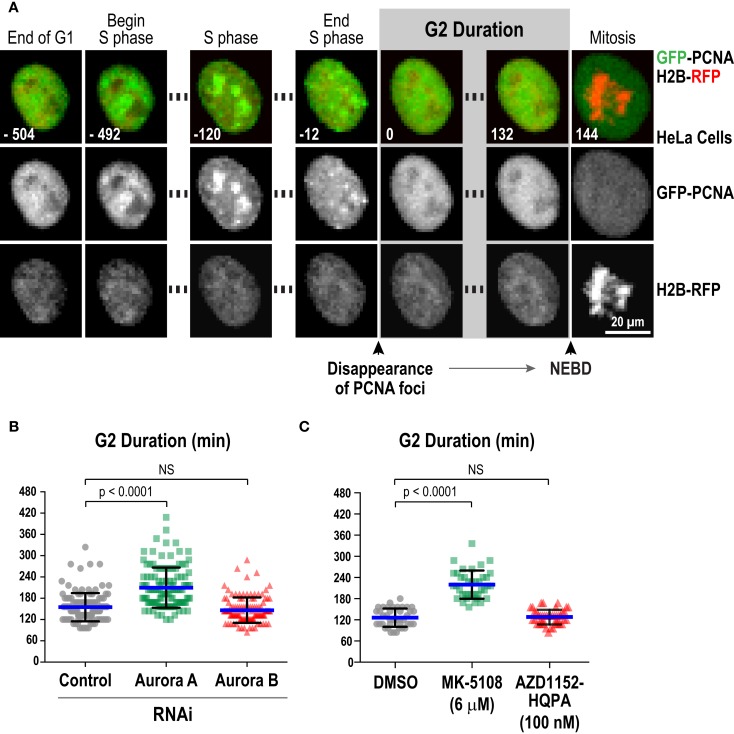
**Measurement of G2 duration provides a specific readout for Aurora A kinase activity**. **(A)** Images from a timelapse sequence of HeLa cells stably expressing GFP-PCNA; H2B-RFP. Gray scale images for the two separate channels are located below each colored merge. G2 duration is measured as the time from dissolution of PCNA foci to nuclear envelope breakdown (NEBD). See also Movie S1 in Supplementary Material.**(B)** Analysis of G2 duration in HeLa cells following knockdown of Aurora A or Aurora B by RNAi. Blue lines indicate the mean; black error bars are the SD. *p*-values are from unpaired *t*-tests. **(C)** Analysis of G2 duration in HeLa cells using selective Aurora A versus Aurora B inhibition, with MK-5108 (6 μM) and AZD1152-HQPA (100 nM), respectively. Blue lines indicate the mean; black error bars are the SD. *p*-values are from unpaired *t*-tests.

We next characterized the effect of selected inhibitors in single-cell live imaging assays, which provide high resolution, dynamic assessment of kinase function in a cellular context. For this purpose, the key challenge was to identify a specific readout for each kinase. For Aurora B, cytokinesis failure is a robust and well-established cellular phenotype of inhibition, which we confirmed with the four Aurora B-specific inhibitors (Figure S3 in Supplementary Material). However, for Aurora A, a specific quantifiable live imaging readout has been lacking. Prior work in *Xenopus* egg extracts (87), *Caenorhabditis elegans* embryos (88), and mammalian cells (89, 90) has suggested a role for Aurora A in controlling the kinetics of mitotic entry. Entry into mitosis, as defined by nuclear envelope breakdown (NEBD), is delayed in the absence of Aurora A. To quantitatively monitor this function of Aurora A in living cells, we employed an assay in which eGFP-tagged PCNA (GFP-PCNA) and mRFP-tagged histone H2B (H2B-RFP) are co-expressed and imaged in a cell population (91). PCNA concentrates in foci known as replication factories in S-phase (Figure 6A; Movie S1 in Supplementary Material) and the time interval from dissolution of PCNA foci to NEBD serves as a measure of G2 duration in living cells (Figure 6A; Movie S1 in Supplementary Material) (91–93). Using this assay in HeLa cells, we found that depletion of Aurora A, but not Aurora B, by RNAi significantly increased G2 duration (Figure 6B). We next measured G2 duration in HeLa cells following treatment with MK-5108 and AZD1152-HQPA, at concentrations that selectively eliminate labeling of pLATS2(Ser 83) or pH3(Ser 83), respectively (6 μM for MK-5108 and 100 nM for AZD1152-HQPA; Figure 4A). In agreement with the RNAi analysis, MK-5108, but not AZD1152-HQPA, significantly increased G2 duration (Figure 6C). Thus, measurement of G2 duration using the GFP-PCNA; H2B-RFP imaging assay provides a specific functional readout for Aurora A activity in living cells.

**Figure 7:**
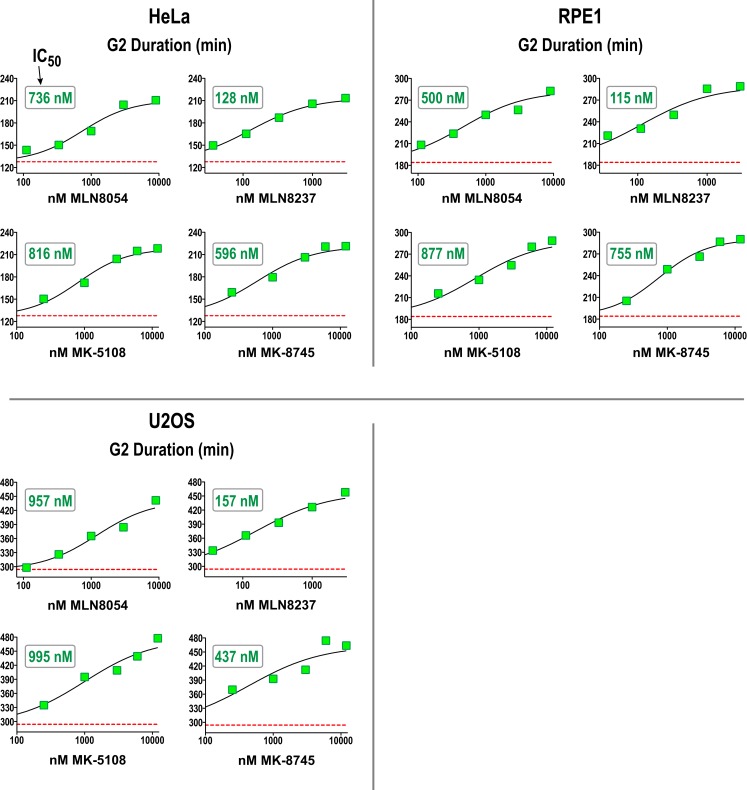
**Dose–response analysis of G2 duration with inhibitors specifically targeting Aurora A in 3 cell lines**. G2 duration (in minutes), measured as in Figure 6, for the indicated inhibitors and cell lines. Each point represents the mean of 40–100 cells from two sets of measurements. See Figure S4 in Supplementary Material for scatter plots showing all measurements. IC_50_ values shown on the graphs were determined by computing dose–response curves using a four-parameter, variable slope fit in GraphPad Prism.

We next performed a dose–response analysis of the inhibitors developed to target Aurora A in the G2 duration assay in HeLa, RPE1, and U2OS. The results are shown in Figure 7 (and Figure S4 in Supplementary Material) and highlight that measurement of G2 duration with this assay provides a sensitive and dose-responsive measure for Aurora A activity in cells. The concentrations where G2 duration was maximally extended by Aurora A inhibitors tracked well with the concentrations at which pLATS2(Ser 83) labeling was eliminated (see Table 3 and text below). This concordance between distinct cell-based assays confirms that each assay specifically monitors Aurora A activity and gives us confidence that the inhibitor characterization performed using them is providing an accurate picture of efficacy in a cellular context.

**Table 3:** **Cellular inhibitory potencies of the Aurora inhibitor panel**.

Aurora kinase inhibitor	Cell line	Assay			
		Substrate-phosphorylation		G2/M	Cytokinesis
		pLATS2 (Ser83)	pHistone H3 (Ser28)		
		IC_50_ (nM)	IC_50_ (nM)	IC_50_ (nM)	IC_50_ (nM)
VX-680	HeLa	150	45	nd	nd
	RPE1	nd	nd	nd	nd
	U2OS	nd	nd	nd	nd
MK-5108	HeLa	610	NE@9 μM	816	nd
	RPE1	1800	80% Inh @15 μM	877	nd
	U2OS	1200	20% Inh @15 µM	995	nd
MK-8745	HeLa	610	NE@9 µM	596	nd
	RPE1	2300	NE@15 µM	755	nd
	U2OS	1700	NE@15 µM	437	nd
MLN8054	HeLa	290	2900	736	nd
	RPE1	720	880	500	nd
	U2OS	800	3300	957	nd
MLN8237	HeLa	37	160	128	nd
	RPE1	130	130	115	nd
	U2OS	92	210	157	nd
Genentech Aurora Inhibitor 1	HeLa	490	NE@9 μM	nd	nd
	RPE1	nd	nd	nd	nd
	U2OS	nd	nd	nd	nd
ZM447439	HeLa	NE@3 μM	419	nd	615
	RPE1	nd	nd	nd	1315
	U2OS	nd	nd	nd	613
AZD1152-HQPA	HeLa	NE@100 nM	5	nd	4
	RPE1	NE@200 nM	25	nd	23
	U2OS	NE@200 nM	12	nd	20
Hesperadin	HeLa	NE@100 nM	14	nd	14
	RPE1	nd	nd	nd	47
	U2OS	nd	nd	nd	17
GSK1070916	HeLa	NE@100 nM	6	nd	6
	RPE1	NE@200 nM	15	nd	21
	U2OS	NE@200 nM	8	nd	6

*NE, no effect; nd, not determined*.

### Immunoblotting-Based Assessment of Inhibitor Potency and Specificity

**Figure 8:**
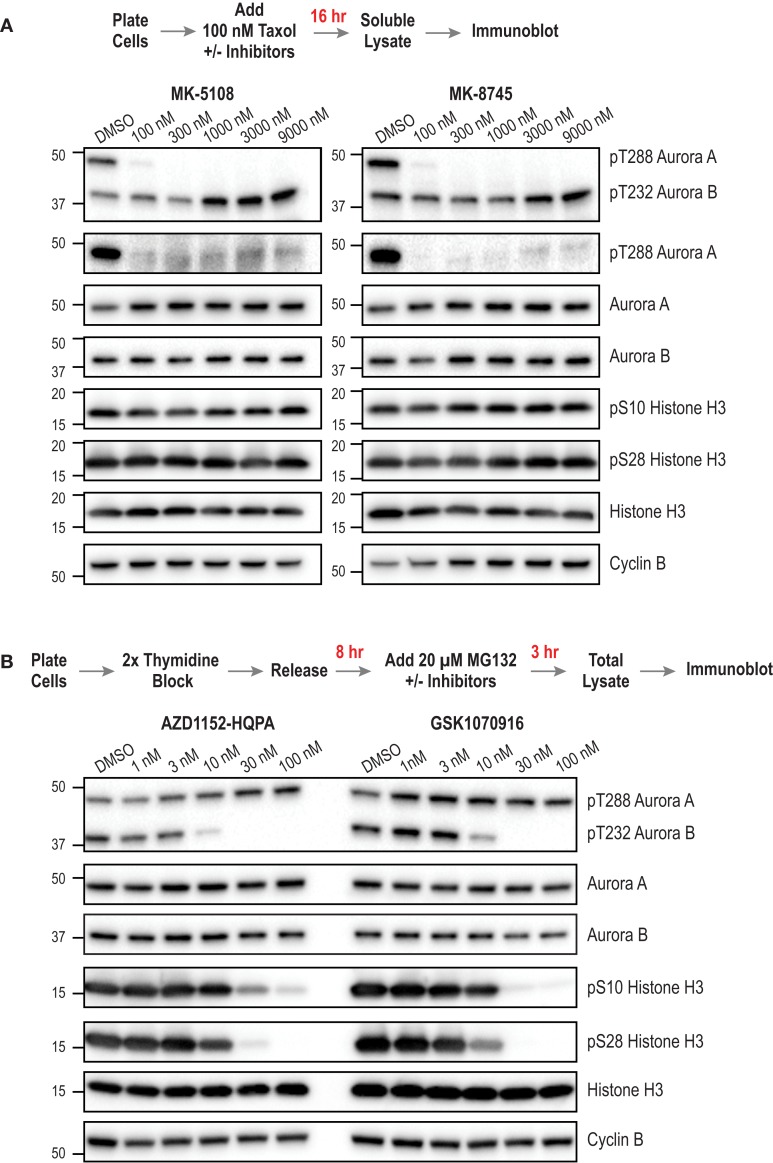
**Immunoblotting of activation loop phosphorylation of Aurora A/B and of pH3(Ser 28) and pH3(Ser 10) in HeLa cells following inhibitor treatments**. **(A)** Analysis of MK-5108 and MK-8745. The targets blotted are indicated on the right; MW markers (in kD) are on the left. The topmost blot is with an antibody that recognizes the phosphorylated activation loops of both Aurora A and Aurora B. The protocol used to prepare mitotic taxol-arrested lysates is summarized above the blots. **(B)** Analysis of AZD1152-HQPA and GSK1070916 as in **(A)**. The only difference is the protocol used to prepare the mitotic lysate; as Aurora B inhibition overrides taxol-based mitotic arrest, a synchronization procedure followed by proteasome inhibition, schematized above the blots, was used. Blots were repeated in at least two independent experiments, which were highly consistent; blot sets from one representative experiment are shown. For **(B)**, see Figure S6 in Supplementary Material for an independent experiment performed at a higher top dose (300 nM).

Next, we sought to compare the inhibitor potency and specificity measurements obtained using the cellular assays to more proximal markers of cellular activity – namely phosphorylation of Aurora A and Aurora B. We developed methods to monitor kinase phosphorylation by immunoblotting because we found it to have higher signal-to-noise and greater consistency than immunofluorescence. For this analysis, we focused on the four inhibitors with the best overall cellular profiles as Aurora A-selective (MK-5108, MK-8745) or Aurora B-selective (AZD1152-HQPA and GSK1070916). After treating cells with different concentrations of these inhibitors, we performed Western blotting for eight targets for which commercial antibodies are available – pAuroraA(Thr 288), pAuroraA(Thr 288)/pAuroraB(Thr 232)/pAuroraC(Thr 198), total Aurora A, total Aurora B, pH3(Ser 28), pH3(Ser 10), total H3, and Cyclin B. We did not assess pLATS2(Ser 83), because the antibody used for immunofluorescence did not work well for immunoblots. The specific antibodies used for immunoblotting were selected based on extensive testing, employing both siRNA depletion (to assess specificity; Figure 3A; Figure S5 in Supplementary Material) and inhibitor treatments (to confirm detection of phospho-epitopes; Figures 8A,B); see Table S3 in Supplementary Material for descriptions and supplier information.

For analysis of the Aurora A-selective compounds, MK-5108 and MK-8745, we employed the protocol outlined in Figure 8A, based on taxol-induced mitotic checkpoint arrest. For analysis of Aurora B-selective inhibitors, AZD1152-HQPA and GSK1070916, we modified a previously described protocol [outlined in Figure 8B; (94)] whose design reflects the fact that Aurora B inhibition overrides taxol-induced arrest (44, 45). To ensure a fair comparison between different conditions, we immunoblotted Cyclin B to confirm that a similar number of mitotic cells were present in the analyzed lysates, in addition to blotting for total H3 as a general loading control. While optimizing the immuoblotting assays, we found that pAuroraA(Thr 288) exhibited low solubility compared to total Aurora A, pAuroraB(Thr 232), or total Aurora B in a typical cell lysis buffer containing non-ionic detergent; only with extensive sonication were we able to solubilize the pAuroraA(Thr 288) signal. This observation suggests that autophosphorylated Aurora A is associated with insoluble cytoskeletal elements, possibly microtubules or centrosomes. From a technical perspective, this observation highlights the importance of employing lysate preparation conditions that properly solubilize pAuroraA(Thr 288) in order to avoid false negative results and/or overestimates of inhibitor potencies. In situations where changes in cell number/viability are not expected (obviating the need to normalize loading by measuring lysate protein concentrations), samples could be prepared by lysing cells directly with SDS gel sample buffer.

The immunoblotting analysis of autophosphorylated Aurora A, pH3(Ser 28), and pH3(Ser 10), confirmed the specificity of MK-5108 and MK-8745 for Aurora A and AZD1152-HQPA and GSK1070916 for Aurora B. At concentrations of MK-5108 and MK-8745 that completely eliminate Thr 288 phosphorylation (and pLATS2(Ser 83) signal in the fixed immunofluorescence assay), there is no effect on pH3(Ser 10), pH3(Ser 28), or pAuroraB(Thr 232) (Figure 8A). Reciprocally, AZD1152-HQPA and GSK1070916 eliminated pH3(Ser 10), pH3(Ser 28), and pAuroraB(Thr 232) at concentrations that did not affect pAuroraA(Thr 288) (Figure 8B).

For AZD1152-HQPA and GSK1070916, there was strong correspondence between the concentration-dependent effects on H3 and Aurora B phosphorylation with those observed in the immunofluorescence and cytokinesis assays (Figures 4A and 8B; Figure S3 in Supplementary Material; Table 3). However, for MK-5108 and MK-8745, complete loss of Aurora A Thr 288 phosphorylation was observed at significantly lower inhibitor concentrations than those necessary for full efficacy in the LATS2 phosphorylation and G2 duration assays [(IC_50_ pAuroraA(Thr 288) <<100 nM versus IC_50_ pLATS2 (Ser 83) and G2 duration: ~ 600-800 nM)] (Figures 4A, 7 and 8A; Table 3). There are three potential explanations for this difference. First, the immunoblotting of activation loop phosphorylation, at least for Aurora A under the conditions employed here, may have a significantly lower dynamic range than the two cell-based assays. Second, the phosphatases that remove pAuroraA(Thr288) (27, 95, 96) may be more efficient than those that reverse pLATS2(Ser 83) and the Aurora A phosphorylation target(s) that contribute to G2 duration control. Third, this difference may arise from cellular Aurora A existing in multiple active but biochemically distinct forms, as proposed previously (28, 32). Recent biochemical data indicate that Thr 288 phosphorylation is not a prerequisite for Aurora A kinase activity if Aurora A is bound to TPX2 (28, 32). Further, our *in vitro* studies indicate that Aurora A/TPX2 is more difficult to inhibit than the free enzyme. Therefore, if LATS2 phosphorylation and mitotic entry kinetics are dependent upon Thr 288 unphosphorylated but active pools of Aurora A (bound to activators), sole assessment of Thr 288 phosphorylation may provide a misleading view of inhibitor potencies. Additional studies will be required to explore these possibilities.

Regardless of the underlying reasons, our data highlight that if a pAuroraA(Thr 288) immunoblot was employed with pH3 immunofluorescence/immunoblots to characterize inhibitor effects, one would conclude that MK-5108 and MK-8745 completely block cellular Aurora A activity at much lower concentrations than we measure for the pLATS2(Ser 83) immunofluorescence and live cell G2 duration assays, and that MK-5108 and MK-8745 have significantly greater selectivity in a cellular context than is actually the case. Thus, we caution on relying exclusively on immunoblotting, especially with activation loop phosphorylation antibodies for Aurora A, to measure inhibitor potency and specificity. Instead, we recommend performing quantitative fixed or live imaging-based analysis of kinase activity, and complementing with immunoblotting.

### Synthesis of Biochemical and Cellular Profiling Data to Identify the Best Aurora A- and B-Selective Inhibitors

Differences in ATP concentrations (biochemical – micromolar; cellular milieu – millimolar) as well as compound solubility/stability, binding to serum proteins in media, and cellular penetration make direct correlation of biochemical and cellular inhibitor potencies difficult (97). However, the Aurora A/B selectivity ratios derived from our *in vitro* and *in vivo* data can be compared to prioritize inhibitors. In the biochemical assays, AZD1152-HQPA, ZM447439, Hesperadin, and GSK1070916 all exhibit >30-fold selectivity for Aurora B/INCENP^783–918^ over Aurora A and Aurora A/TPX2^1–43^ (Table 2). This selectivity is recapitulated in the cellular analysis. Complete inhibition of Aurora B(Thr 232) and H3(Ser 28) phosphorylation (as well as blockade of cytokinesis) can be achieved with each of these compounds in the absence of any effect on Aurora A(Thr 288) or LATS2(Ser 83) phosphorylation (Table 3). So, which of these compounds is the best for cell biology experiments? Hesperadin is potent and specific but appears to be unstable under long-term live imaging conditions (see legend of Figure S3 in Supplementary Material). Within the quinazoline class of compounds (Figure 1), AZD1152-HQPA is preferable as it is approximately two orders of magnitude more potent *in vitro* and *in vivo* than ZM447439, from which it was derived. How then do AZD1152-HQPA and GSK1070916 compare? Although both are selective, AZD1152-HQPA exhibits significantly greater preference for Aurora B/INCENP^783–918^ when compared to either Aurora A or Aurora A/TPX2^1–43^*in vitro* (Table 2). Consistent with this, immunoblotting revealed that, while both compounds eliminated Aurora B activity at <100 nM without affecting pAuroraA (Thr 288) (Figure 8), at 300 nM GSK1070916 inhibited Aurora A activity whereas AZD1152-HQPA did not (Figure S6 in Supplementary Material). In addition, when profiled against 363 human kinases (including Aurora A/B/C) at 100 nM concentration (Table S4 in Supplementary Material), both compounds exhibited high Aurora kinase-specificity but AZD1152-HQPA was slightly superior. Aurora B was the only enzyme inhibited ≥65% of control by AZD1152-HQPA whereas Aurora A and B as well as DDR1 are inhibited by GSK1070916 at or above this threshold (Table S4 in Supplementary Material). Therefore, we believe that AZD1152-HQPA is the current best choice for an Aurora B-selective inhibitor, and recommend use of GSK1070916 for confirmatory follow-up studies (*see below*).

In terms of the Aurora A inhibitors, MLN8054 and MLN8237 exhibit only modest selectivity *in vitro* and *in vivo* (Tables 2 and 3). Genentech Aurora Inhibitor I is cytotoxic within the range of concentrations required for full inhibition of Aurora A activity (Figure 4D). In the fixed and live imaging cell-based assays, the two Merck compounds were similar with MK-5108 being slightly more potent (Table 3) and MK-8745 being more selective in both RPE1 and U2OS cells (Figure 5; Table 3). By kinome profiling analysis, MK-8745 was significantly more Aurora kinase-specific. At 100 and 500 nM, MK-5108 inhibited 32 and 75 kinases (including the Aurora kinases), respectively, at >65% of control; Polo-like kinase 4 was the only cell cycle-related kinase affected (Table S4 in Supplementary Material). In contrast, MK-8745 at 500 nM only inhibited 16 kinases at >65% of control (Aurora A, AXL, BRK, DDR1, EphA6, GSK3α/β, IRAK1, JNK1, LKB1, ROS1, Trk A/B/C, TYRO3, YES). Thus, based on its Aurora and off-target selectivity, we believe that MK-8745 is the current best commercially available Aurora A-selective inhibitor for cellular studies.

### The Structure of the Aurora A Kinase Domain Bound to MK-5108 Reveals Features Underlying Potency and Specificity

**Figure 9:**
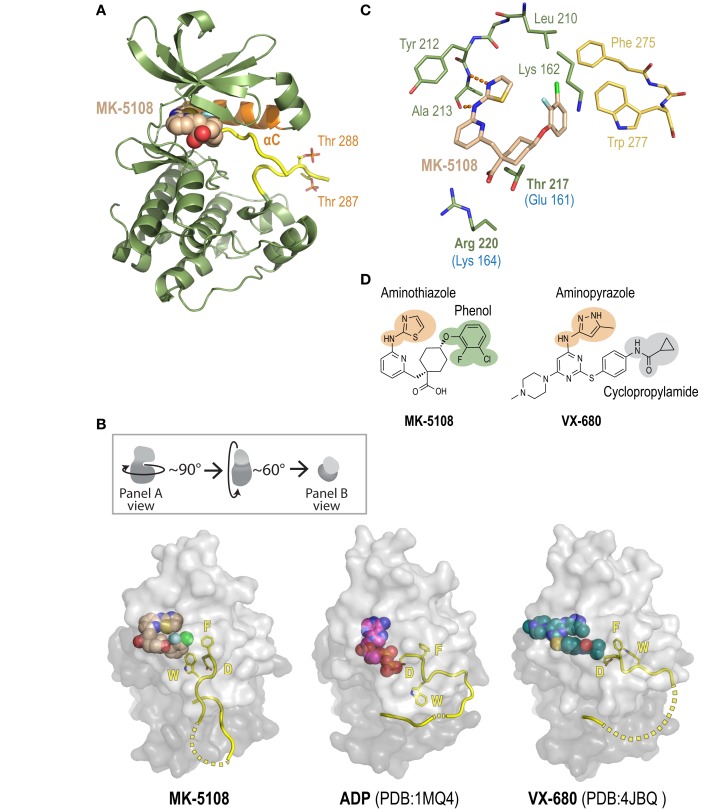
**A crystal structure of the Aurora A/MK-5108 complex reveals unique features compared to previous Aurora A-inhibitor complex structures**. **(A)** The complex structure with the Aurora A kinase domain depicted as a ribbon diagram and MK-5108 as a spacefilling model. The majority of the kinase domain is colored green except for the activation loop, which is colored yellow, and the αC helix, which is colored orange. The phosphate groups on Thr 288 and Thr 287 are highlighted. **(B)** Comparison of Aurora A/MK-5108 structure to previously determined ADP-bound (PDB:1MQ4) and VX-680-bound (PDB: 4JBQ) structures. The orientation of the kinase domains relative to that in **(A)** is described in the inset. For all three structures, beyond the activation loop (yellow cartoon – dashed lines indicate disordered regions) only the protein surface is shown (light gray – N-terminal lobe, dark gray – C-terminal lobe). Asp 274 (D) and Phe 275 (F) of the DFG motif, as well as Trp 277 (W) are depicted. **(C)** Detailed view of the key contacts between MK-5108 and Aurora A residues. Hydrogen bonds between the aminothiazole of MK-5108 and Ala 213 are shown as orange dashes. MK-5108 interacts with the side chains of two residues which are different between Aurora A and B (Aurora A: Thr 217 and Arg 220; Aurora B: Glu 161 and Lys 164; the Aurora B residues are indicated in blue text below the corresponding Aurora A ones in the figure). See also Table S6 in Supplementary Material. For clarity, the main chain atoms of Lys 162, Thr 217 and Arg 220 as well as the side chain atoms of Glu 211 are not shown. **(D)** The chemical structures of MK-5108 and VX-680 with specific moieties highlighted.

To gain insight into the remarkable biochemical potency and Aurora A-selective nature of the MK-8745/MK-5108 class of compounds, we determined the 2.2 Å X-ray crystal structure of the human Aurora A kinase domain bound to MK-5108 (Figure 9A; Table S5 and Figure S7 in Supplementary Material). In the inhibitor complex, the Aurora A kinase domain adopts an inactive conformation in which both the αC helix (*orange*; Figure 9A) and particularly the activation loop (*yellow*; Figure 9A) are improperly positioned for catalysis (Figure 9B – compare MK-5108-bound versus ADP-bound structures). Electron density maps indicate that both Thr 287 (which adopts two alternate conformations; only one is illustrated) and Thr 288 are phosphorylated, showing that the inhibitor is able to interact with the activated form of the enzyme (Figure 9A). Consistent with its action as an ATP-competitive inhibitor (56), MK-5108 inserts itself into the nucleotide-binding pocket between the two lobes of the kinase in the same orientation as its parent compound VX-680 (Figures 9A,B).

The picomolar affinity of MK-5108 for Aurora A is explained by the extensive polar and van der Waals interactions it forms with 22 residues throughout the active site (Figure 9C, Table S6 in Supplementary Material). The aminothiazole moiety (Figures 9C,D) is located adjacent to the gatekeeper residue Leu 210 enabling it to form two hydrogen bonds with the main chain amide nitrogen and carbonyl of Ala 213 within the hinge region (Figure 9C), thereby making it functionally analogous to the aminopyrazole moiety of VX-680 (Figure 9D). The 2-fluoro, 3-chlorophenol on the opposite end of the inhibitor (Figure 9D) packs against the side chains of the catalytic lysine (Lys 162), precluding its active conformation, as well as against Phe 275 of the DFG motif (Figure 9C). This interaction stabilizes a flipped, inactive conformation of the DFG motif that is intermediate between the active “DFG-in” state (Figure 9B – ADP-bound) (98) and the canonical “DFG-out” conformation (99). This conformation is distinct from the distorted conformation in the VX-680 Aurora A complex (Figure 9B – VX-680-bound) (74), and the “DFG-up” conformation linked to MLN8054 binding (59, 71, 75), and resembles that of Aurora A bound to adenosine (PDB: 1MUO) (100). Importantly, the 2-fluoro, 3-chlorophenol moiety of MK-5108 forms a likely highly energetically favorable edge-face aromatic pi stacking interaction with the indole of Trp 277 (Figures 9C,D). Because of the major differences in its chemical structure in this region (Figure 9D), VX-680 only forms hydrophobic contacts with Phe 275 (via its cyclopropylamide) and not Trp 277 (Figure 9B). The interaction between MK-5108 and the side chain of Trp 277 has the effect of “pinning down” the activation loop at its N-terminal end and disfavors its adoption of an active conformation (Figure 9B). We note that the phosphates on Thr 287 and Thr 288 form hydrogen bonds with the side chains of His 187 and Lys 250, respectively, from a symmetry-related molecule. However, since residues 281–285 are disordered (Figure 9B), we believe that residues 277–280 should not be constrained by these contacts, and that their positioning is a consequence of inhibitor binding. The inactive conformation of the activation loop that we describe here is, to our knowledge, unique among all known human Aurora A kinase domain – inhibitor complex structures. The conformations of the active site and activation loop residues stabilized by MK-5108 binding are distinct from those favored by TPX2 binding, which likely explains the reduced affinity of MK-5108 for the Aurora A/TPX2^1–43^ complex.

The crystal structure of the MK-5108/Aurora A complex also suggests a potential explanation for the selectivity of this inhibitor. MK-5108 interacts with the side chains of two (Thr 217 and Arg 220) of the four residues in the vicinity of the active site that differ between Aurora A and B (Aurora A: Ala 141, Leu 215, Thr 217 and Arg 220; Aurora B: Lys 85, Arg 159, Glu 161, Lys 164) (Figure 9C; Table S6 in Supplementary Material). Although the electron density for Aurora A Arg 220 is weak (indicative of mobility), the positively charged guanidinium of this residue is located close enough to the negatively charged MK-5108 carboxylate to form favorable electrostatic interactions (Figures 9C,D). However, the equivalent Aurora B residue is a lysine (Lys 164), which should also be able to form the same types of interactions. In contrast, the side chain of Thr 217 is wedged in between the carboxylate and the cyclohexyl ring of MK-5108 (Figures 9C,D). This tight fit would not be possible with the equivalent Glu 161 in Aurora B, likely significantly reducing binding affinity. Integrated mutagenesis, biochemical and structural studies of MLN8054 and Genentech Aurora A Inhibitor I (which both pack against Thr 217) have indicated that Thr 217 contributes heavily to the selectivity of these compounds (59, 71, 75). Equivalent efforts with MK-5108 and MK-8745 (which, by modeling, is predicted to bind in a highly similar manner as MK-5108 to the Aurora A active site) will be necessary to test if Thr 217 is the central determinant of Aurora A/B selectivity for these compounds, as suggested by our structural analysis.

## Discussion

The current tool chest of Aurora inhibitors is the product of extensive chemical optimization in the pursuit of suitable clinical candidates, rather than optimal inhibitors for cell biology studies. As a consequence, careful comparison of these inhibitors in parallel biochemical and cell-based assays has been lacking. The systematic profiling data presented here should provide a resource for future studies employing these compounds. Based on our results, we provide the following ‘good practice’ guidelines with respect to their use:

(1) pH3(Ser 28) and pLATS2(Ser 83) labeling, which we validate as specific cellular readouts for Aurora B and A, respectively, provides a convenient and robust means to characterize existing and newly developed Aurora kinase inhibitors, and should be used prior to any detailed functional analysis conducted with this class of compounds. Inhibitor sensitivity can be modulated by biological factors (such as kinase expression levels) or technical factors (such as changes in serum/media and growth conditions) (97). Hence, dose–response analysis employing the pH3(Ser 28)/pLATS2(Ser 83) labeling assay is particularly important when extending inhibitor use to new cell lines not analyzed here. While we present a large dataset for HeLa, RPE1, and U2OS cells that should serve as a benchmark for future studies, we still recommend performing a dose–response even when using these three lines given inter-lab variability. The goal of such preliminary analysis should be to identify the *minimum* concentration that achieves complete inhibition of the relevant marker without affecting the other. We strongly advise against the “more is better” urge as unnecessarily high doses will likely lead to loss of specificity and potential unanticipated off-target effects.
(2) AZD1152-HQPA is a highly potent, selective, and efficacious Aurora B inhibitor and the best current choice for targeting this kinase. While AZD1152-HQPA does not exhibit any obvious effects against any of the other kinases that we tested, this profiling exercise was not exhaustive. Further, there is relatively little published about binding of this compound to non-kinase proteins, and even very well-characterized molecules can have unexpected off-target effects. For example, recent studies have revealed that the commonly used Plk1 inhibitor, BI-2536, and several other known kinase inhibitors, are potent inhibitors of BET bromodomain proteins (101–103). Therefore, we highly recommend that any results from studies using AZD1152-HQPA be corroborated with GSK10701916, which likely has a different off-target profile based on its unrelated chemical structure. Indeed, this strategy of using structurally distinct compounds with common mechanisms should be applied when using any of the inhibitors analyzed here and when performing chemical cell biology studies in general.
(3) MK-8745 represents the best current commercially available option for selective and potent Aurora A inhibition. We note, however, that the lowest concentration of MK-8745 that is necessary to maximally inhibit Aurora A in cells is >100-fold higher than the lowest concentration of AZD1152-HQPA that is necessary to fully inhibit Aurora B. Hence, based on current chemical biology standards [on-target cellular activity <1 μM; (104, 105)], AZD1152-HQPA is an ideal chemical tool whereas MK-8745 is not. In addition, validation of any findings with MK-8745 with an orthogonal chemical scaffold is not currently straightforward. If only partial inhibition of Aurora A is required, MLN8054 (and possibly MLN8237) could be used but only under carefully controlled circumstances. Alternatively, MK-5108 could be used but this is also not optimal given its chemical similarity to MK-8745. In the recent literature, at least five classes of compounds with Aurora A-selective behavior (which are not commercially available or only became available near the end of this study) have been reported (106–110). Assessment of these inhibitors should reveal if one or more of them can be paired with MK-8745 for analysis of Aurora A function in cellular experiments.
(4) Immunoblotting of activation loop phosphorylation should not be used in isolation to estimate inhibitor potency and specificity. As we show here, immunoblotting with pAuroraA(Thr 288) antibodies suggests significantly higher Aurora A inhibitor potency than is observed in validated fixed and live imaging-based cellular assays. Consequently, if only immunoblotting were performed, one could overestimate not just potency but also selectivity for Aurora A versus Aurora B. We recommend that the fixed or live imaging-based cellular assays described here be employed first, with immunoblotting serving as confirmation. The imaging-based cellular assays also have the advantage of revealing potential off-target effects, such as the toxicity of the Genentech Aurora Inhibitor 1 reported here.

Our analysis highlights that, while highly selective and potent tools for Aurora B inhibition are readily available, there is significant room for improved small molecule inhibitors of Aurora A. Part of the challenge in targeting Aurora A likely arises from its multiple activation mechanisms, which makes uniformly inhibiting the different active states of the kinase difficult. A second limitation is the prior lack of a consistent and rigorous assay paradigm for Aurora A activity in a cellular context – as we show here, immunoblotting of activation loop phosphorylation can be misleading when compared to other kinase activity readouts – a fact that can be rationalized by recent findings that activation loop phosphorylation is not essential for high levels of kinase activity in the presence of an activator such as TPX2. Our findings suggest new avenues to help address the challenge of developing a more potent and highly selective Aurora A inhibitor. First, the pLATS2(Ser 83) and the G2 duration assays provide independent, robust, and dose-responsive cellular readouts that specifically report on Aurora A but not Aurora B activity. These assays could be used for optimization of novel classes of Aurora A inhibitors in a cell-based context, analogous to the strategy we employed recently to develop a Plk4 inhibitor, centrinone, that prevents centriole duplication (92). The target specificity of centrinone was confirmed through the extensive use of an engineered inhibitor-resistant mutant. Analogous approaches could also be applied using previously described inhibitor-resistant Aurora kinase mutants (75, 111, 112). Further, the crystal structure of the MK-5108/Aurora A kinase domain complex we determined, which revealed a previously unobserved protein conformation and active site interactions, could be used to generate more potent versions of MK-5108/MK-8745 and potentially design new molecules as well. Given the renewed interest in Aurora A as a drug target based on the recently discovered role of Aurora A in controlling c-Myc protein levels in cancers such as neuroblastoma (113, 114), new inhibitor discovery efforts leveraging the approaches described here could aid not only in developing better tools for cell biology experiments but also in fully realizing the therapeutic potential of inhibiting Aurora A.

## Materials and Methods

### Inhibitors and Antibodies

Inhibitors and antibodies used in this study are described in Table S1 and S3 in Supplementary Material, respectively.

### Kinase Assays

For the Aurora A assays, purified full-length human Aurora A (Millipore) was diluted to ~0.8 nM (based on enzyme activity) in 7.5 μL of a buffer containing 50 mM Tris (pH 7.5), 150 mM NaCl, 270 mM sucrose, 0.03% Brij 35, and 1 mM DTT in Corning #4512 white 384-well plates. Inhibitors arrayed in dose–response were added from DMSO stocks using a V&P 384-pintool head mounted on a Beckman Multimek chassis. Reactions were then initiated via the addition of 7.5 μL of a buffer containing 50 mM Tris (pH 7.5), 20 mM MgCl_2_, 1 mM DTT, 0.2 mg/ml BSA, 70 μM ATP, and 800 μM Kemptide (amino acid sequence: LRRASLG (InnoPep)) using a NSX-384 384-channel liquid handler (Nanoscreen), and allowed to proceed for 2 h at 25°C. The final reaction buffer contained 50 mM Tris (pH 7.5), 10 mM MgCl_2_, 75 mM NaCl, 135 mM sucrose, 0.015% Brij 35, 1 mM DTT, 0.1 mg/mL BSA, 35 μM ATP, and 400 μM Kemptide. The final [ATP] in the reaction mix (35 μM) is at the *K*_m_(ATP) for Aurora A. Detection using a 5 μL aliquot of each reaction was performed with ADP-Glo™ reagents (Promega), following the manufacturer’s instructions, in PerkinElmer #6008281 plates. Luminescence was measured on an Infinite M1000 plate reader (Tecan). Data were fit using a 4-parameter, variable slope fit in Prism (GraphPad), and *K*_i_s were calculated from IC_50_ data using the equation in Figure 2B.

For the Aurora A/TPX2^1–43^ assays, purified full-length human Aurora A (Millipore) was diluted to ~0.8 nM (based on enzyme activity) in 7.5 μL of a buffer containing 80 nM TPX2^1–43^ (InnoPep), 50 mM Tris (pH 7.5), 150 mM NaCl, 270 mM sucrose, 0.03% Brij 35, and 1 mM DTT in Corning #4512 white 384-well plates. The TPX2^1–43^ concentration was determined using a calculated molar extinction coefficient (280 nm) of 8480 M^−1^ cm^−1^. Inhibitors arrayed in dose–response were added from DMSO stocks using a V&P 384-pintool head mounted on a Beckman Multimek chassis. Reactions were then initiated via the addition of 7.5 μL of a buffer containing 50 mM Tris (pH 7.5), 20 mM MgCl_2_, 1 mM DTT, 0.2 mg/ml BSA, 6 μM ATP, and 1,200 μM Kemptide using a NSX-384 384-channel liquid handler (Nanoscreen), and allowed to proceed for 1 h at 25°C. The final reaction buffer contained 50 mM Tris (pH 7.5), 10 mM MgCl_2_, 75 mM NaCl, 135 mM sucrose, 0.015% Brij 35, 1 mM DTT, 0.1 mg/mL BSA, 3 μM ATP, and 600 μM Kemptide. The final [ATP] in the reaction mix (3 μM) is at the *K*_m_(ATP) for Aurora A/TPX2^1–43^. At the final concentration of 40 nM, TPX2^1–43^ is >10 times the concentration required to achieve half-maximal activation of Aurora A under these reaction conditions (3 nM) and the previously reported *K*_d_ of TPX2^1–43^ [2.3 nM (70)]. Detection, measurement, and data analysis were performed as described above.

For the Aurora B/INCENP^783–918^ assays, purified full-length human Aurora B/INCENP^783–918^ (SignalChem) was diluted to ~0.5 nM (based on enzymatic activity) in 12 μL of a buffer containing 31.25 mM Tris (pH 7.5), 12.5 mM MgCl_2_, 93.75 mM NaCl, 168.75 mM sucrose, 0.0125% Tween 20, 0.625 mM DTT, 0.1875 mg/mL BSA, and 500 μM Kemptide in Corning #3657 clear 384-well plates. Inhibitors arrayed in dose–response were added from DMSO stocks using a V&P 384-pintool head mounted on a Beckman Multimek chassis. After 15 min at 25°C, reactions were initiated via the addition of 3 μL of 50 μM ATP using a NSX-384 384-channel liquid handler (Nanoscreen), and allowed to proceed for 1 h at 25°C. The final reaction buffer contained 25 mM Tris (pH 7.5), 10 mM MgCl_2_, 75 mM NaCl, 135 mM sucrose, 0.01% Tween 20, 0.5 mM DTT, 0.15 mg/mL BSA, 10 μM ATP, 400 μM Kemptide. The final [ATP] in the reaction mix (10 μM) is at the *K*_m_(ATP) for Aurora B/INCENP^783–918^. Detection, measurement and data analysis were performed as described above.

Radiometric assay-based kinome profiling of AZD1152-HQPA, GSK1070916, MK-5108 and MK-8745 was performed by Reaction Biology Corporation (Malvern, PA, USA) using [ATP] ~ *K*_m_(ATP) for all enzymes.

### Cell Lines

RPE1 (hTERT-immortalized RPE cells) and U2OS osteosarcoma cells were obtained from ATCC. HeLa cervical carcinoma cells were from a laboratory stock. RPE1 cells were maintained in Dulbecco’s modified Eagle’s medium (DMEM)/F12 plus glutamine medium supplemented with 10% fetal bovine serum, 100 U/mL penicillin and 100 μg/mL streptomycin. U2OS and HeLa cells were maintained in DMEM + Glutamax supplemented with 10% fetal bovine serum, 100 U/mL penicillin and 100 μg/mL streptomycin.

For generation of HeLa, U2OS, and RPE1 lines co-expressing H2B-RFP and either GFP-PCNA or YFP-tubulin, cells were infected first with an H2B-RFP expressing retrovirus. A pBABE-puro vector, encoding human histone H2B with mRFP1.3 fused at its C-terminus (H2B-RFP) obtained from the laboratory of Don Cleveland, and pBSK-VSV-G were co-transfected into the packaging cell line GP2-293 (Clontech) using FuGENE HD (Promega). Virus-containing culture supernatant was collected 48 h after transfection and added to the growth medium of cells, followed by addition of Polybrene (Millipore) to 8 μg/ml.

An MGC collection human PCNA cDNA with eGFP fused at its N-terminus (GFP-PCNA) was cloned into pBABE-hygro. A pBABE-bla (blasticidin) vector encoding human alpha 1B tubulin with eYFP fused to its N-terminus (YFP-tubulin) was obtained from the laboratory of Don Cleveland. Virus production and infection of cells previously transduced with H2B-RFP was performed similarly. FACS was used to select cell populations expressing transgenes at moderate levels.

### RNAi

HeLa cells co-expressing GFP-PCNA and H2B-RFP were used for all imaging-based RNAi experiments. ON-TARGETplus SMARTpool siRNAs (GE Healthcare) targeting Aurora A and Aurora B, as well as a non-targeting control pool, were transfected into cells using Lipofectamine RNAiMAX (Thermo Fisher Scientific) at a final concentration of 50 μM in 6-well plates. Five hours after transfection, cells were trypsinized and seeded into a 96-well cycloolefin plate (Greiner) at 10,000 cells/well in fresh medium supplemented with 2.5 mM thymidine. Cells were incubated in the presence of thymidine for 18–20 h, and then 300 ng/mL nocodazole for 6 h. Knockdown was confirmed by immunoblotting with the Aurora kinase antibodies specified in Table S3 in Supplementary Material and an anti-tubulin antibody (DM1A; 1:1000; Sigma).

For fixed analysis to quantify pLATS2(Ser 83), pH3(Ser 10), and pH3(Ser 28) intensities, plates were washed twice with fresh medium and returned to the incubator for 8 h. Cells were then fixed with either 4% PFA = paraformaldehyde (in phosphate-buffered saline, PBS) (for pH3 analysis) or 100% ice-cold methanol (for pLATS2 analysis). The following primary antibodies were used: pLATS2(Ser 83) (see Table S3 in Supplementary Material); pH3(Ser 10) (1:100; Cell Signaling); pH3(Ser 28) (see Table S3 in Supplementary Material). Cells were imaged on the CV7000 spinning disk confocal system (Yokogawa Electric Corporation) using a 40 × 0.95 NA U-PlanApo objective and 2560 × 2160 sCMOS camera with 2 × 2 binning. 5 μm × 2 μm z-sections of 50 fields/well were imaged, with replicate wells per RNAi condition.

For quantification, maximum intensity projections were generated by the CV7000 acquisition software and transferred to ImageJ for analysis. For pLATS2(Ser 83) measurements, the integrated signal from a 10 × 10 pixel box centered on each mitotic spindle pole was measured. For background subtraction, a 10 × 10 pixel box in the cytoplasm was used. Mean values of measurements were normalized to the control RNAi condition. A total of 186–230 measurements from two independent experiments were made. For pH3(Ser 10) and pH3(Ser 28), the DNA signal was used to threshold and define a binary mask, which was transferred to the pH3 channel. The mean intensity of this region was then measured in the pH3 channel. For background subtraction, the masked region was expanded by 20 pixels, and the mean intensity of the peripheral region was used. Mean values of measurements were normalized to the DMSO-treated condition. A total of 88–147 measurements from three independent experiments were made.

For live imaging experiments to measure G2 duration, plates were washed twice with fresh medium, and immediately mounted onto the CV1000 spinning disk confocal system (Yokogawa Electric Corporation). The imaging chamber was maintained at 37°C and 5% CO_2_. Cells were imaged using a 20 × 0.75 NA U-PlanApo objective and 512 × 512 EM-CCD camera with 2 × 2 binning. Twelve fields/well were imaged, with 4 replicate wells per RNAi condition. 3 μm × 2 μm z-sections in the GFP (25% power, 200 ms, 35% gain) and RFP (20% power, 200 ms, 35% gain) channels were captured in each field, at 12-min intervals for 24 h. Quantification was performed as described in the G2 duration assay section (see below).

### Cellular Proliferation Analysis

Eight thousand HeLa cells, 8,000 U2OS cells or 4,000 RPE1 cells were seeded into white 96-well assay plates (Corning #3610) 16 h before inhibitor addition. All inhibitors were diluted in DMSO and added to cells in complete growth media (2× desired concentrations were prepared in complete growth medium and added to wells). After 24 h, relative cell number was measured using ATPLite™ reagent (PerkinElmer) following the manufacturer’s instructions. DMSO-treated cells were used as controls. Two independent experiments with triplicate measurements per condition were performed. Luminescence was measured on an Infinite M1000 plate reader (Tecan).

### Live Cell-Activated Caspase 3/7 Assay

HeLa cells (6,000/well) were seeded in 96-well μCLEAR plates (Greiner) in 100 μL DMEM plus serum, and incubated for 16 h at 37°C and 5% CO_2_. MK-5108 and Genentech Aurora Inhibitor 1 were diluted 1:100 from DMSO stocks into serum-free DMEM and 11 μL of the diluted compound was added to cells. After 24 h 2 μM CellEvent Caspase-3/7 Green Reagent (Life Technologies), and NucBlue Live ReadyProbes Reagent (Hoechst 33342; Life Technologies) were added. Cells were imaged after 60 min on a CV7000 spinning disk confocal system (Yokogawa Electric Corporation) with a 20 × 0.75 NA U-PlanApo objective and 2560 × 2160 sCMOS camera with 2 × 2 binning. The imaging chamber was maintained at 37°C and 5% CO_2_. Six to eight fields/well were imaged, with duplicate wells for each condition. 3 μm × 2 μm z-sections in the blue (40% power, 300 ms, 35% gain) and green (40% power, 300 ms, 35% gain) channels were captured in each field. The apoptotic fraction was calculated by dividing the number of cells fluorescing at 530 nm (corresponding to the cleaved caspase reporter reagent) by the number of nuclei (Hoechst staining). Image analysis was done using the CV7000 image analysis software (Yokogawa Electric Corporation).

### Substrate Phosphorylation Assay

Twelve thousand HeLa cells, 10,000 U2OS cells, or 8,000 RPE1 cells were seeded into 96-well glass-bottom Sensoplates (Greiner) 16 h before inhibitor addition. Prior to seeding, the glass-bottom plates were coated with poly-l-lysine (Sigma). All inhibitors were diluted in DMSO and added to cells in complete growth media (2× desired concentrations were prepared in complete growth medium and added to wells). After 8 h cells were fixed with 4% PFA for 20 min at room temperature. The fixed cells were washed with PBS. For immunostaining, cells were permeabilized and blocked with PBS containing 10% normal donkey serum (Jackson ImmunoResearch) and 0.1% Triton-X100 for 1 h at room temperature. Primary antibodies against phospho-LATS2 (Ser 83), phospho-histone H3(Ser 28), and anti-phospho-MPM2 (see Table S3 in Supplementary Material) were incubated for 1 h at room temperature. Cells were stained with Cy3-conjugated goat anti-rat, Alexa Fluor 488-conjugated goat anti-mouse IgG2b, and Alexa Fluor 647-conjugated goat anti-mouse IgG1 secondary antibodies (see Table S3 in Supplementary Material) and Hoechst 33342 for 1 h at room temperature. Cells were then washed twice with PBS containing 0.1% Triton-X100. Image acquisition in four channels was performed using a CV7000 spinning disk confocal system (Yokogawa Electric Corporation) with a 40 × 0.95 NA U-PlanApo objective and 2560 × 2160 pixel sCMOS camera. Fluorophores (Hoechst 33342, Alexa Fluor 488, Cy3 and Alexa Fluor 647) were excited with 50% laser power for 300 ms and maximum projections of 8–14 μm × 1 μm z-sections were recorded. Fifty fields per well were imaged with quadruplicate wells for each condition. Image analysis was done using the CV7000 image analysis software (Yokogawa Electric Corporation). Between 100 and 1,000 mitotic cells per condition were segmented applying object identification parameters to select for bright MPM2 labeling. Using a nuclear identifier protocol, minimum intensity thresholds were set for pLATS2(Ser 83) and the pH3(Ser 28) signals, and the resulting identified objects were eroded, dilated, and filtered for size by user-defined thresholds. For only the MPM2-positive mitotic cells, the mean fluorescence intensity of the identified pLATS2(Ser 83) and pH3(Ser 28) objects was measured, and the average intensity per cell per well was calculated. The same thresholds were applied for all of the inhibitor-treated samples, which were processed, imaged, and analyzed in parallel with control DMSO-treated cells. Data were fit using a four-parameter, variable slope fit in Prism (GraphPad). Primary and secondary antibody dilutions can be found in Table S3 in Supplementary Material.

### G2 Duration Assay

HeLa, U2OS, and RPE1 cells co-expressing GFP-PCNA and H2B-RFP were seeded into 96-well glass bottom Sensoplates (Greiner) at 10,000 cells/well 16 h before inhibitor addition. Prior to seeding, glass-bottom plates were coated with poly-l-lysine (Sigma). All inhibitors were diluted in DMSO and added to cells in complete growth media (2× desired concentrations were prepared in complete growth medium and added to wells). Movies were acquired on a CV1000 spinning disk confocal system (Yokogawa Electric Corporation) with a 20× U-PlanApo 0.75 NA objective and 512 × 512 EM-CCD camera with 2 × 2 binning. The humidity controlled imaging chamber was maintained at 37°C and 5% CO_2_. Three fields per well were imaged, with duplicate wells for each condition. 3 μm × 2 μm z-sections in the GFP (25% power, 100 ms, 20% gain) and RFP (20% power, 100 ms, 20% gain) channels were captured in each field at 12-min intervals for 24 h. Cells were manually tracked from appearance of GFP-PCNA foci to the beginning of the next mitosis (NEBD). GFP-PCNA foci appear in the nucleus during mid to late S-phase, and the first frame in which these foci are no longer visible was defined as the beginning of G2 phase. Results represent combined measurements of 40-100 cells per condition from two independent experiments. Data were fit using a 4-parameter, variable slope fit in Prism (GraphPad).

### Cytokinesis Assay

HeLa, U2OS, and RPE1 cells co-expressing YFP-α-tubulin and H2B-RFP were seeded into 96-well glass-bottom Sensoplates (Greiner) at 8,000 cells/well 16 h before inhibitor addition. Prior to seeding, glass-bottom plates were coated with poly-l-lysine (Sigma). All inhibitors were diluted in DMSO and added to cells in complete growth media (2× desired concentrations were prepared in complete growth medium and added to wells). Movies were acquired on a CV1000 spinning disk confocal system (Yokogawa Electric Corporation) with a 40× U-PlanApo 0.95 NA objective and 512 × 512 EM-CCD camera. The humidity controlled imaging chamber was maintained at 37°C and 5% CO_2_. Eight fields per well were imaged, with duplicate wells for each condition. 5 μm × 2 μm z-sections in the YFP (25% power, 100 ms, 20% gain) and RFP (20% power, 100 ms, 20% gain) channels were captured in each field at 5-min intervals for 24 h. Cells were manually tracked from mitosis to G1, and the appearance of microtubule midbodies and mono/binucleated daughter cells were analyzed to assess cytokinesis success. Results represent combined measurements of 50-100 cells per condition from two independent experiments. Data were fit using a 4-parameter, variable slope fit in Prism (GraphPad).

### Western Blot Analysis

For Aurora A inhibitors, HeLa cells were seeded into 10 cm dishes and treated with 100 nM taxol and DMSO or compounds in dose–response for 16 h. Cells were harvested at 50–80% confluence and lysed in RIPA buffer supplemented with a protease/phosphatase inhibitor cocktail (Thermo Fisher Scientific) using a Qsonica Q800R sonicator (10 min, 50% amplitude, 15 s on/15 s off). Before loading, concentrations of cleared extracts were normalized using a Bio-Rad Protein Assay (Bio-Rad). For every sample, 25–50 μg protein per lane was run on Mini-PROTEAN gels (Bio-Rad) and transferred to PVDF membranes using a TransBlot Turbo system (Bio-Rad). For primary anti-phospho-Histone H3(Ser 10), anti-phospho-Histone H3(Ser 28), anti-Aurora A, anti-Aurora B, anti-phospho-Aurora A(Thr 288)/Aurora B(Thr 232)/Aurora C(Thr 198), anti-Histone H3, anti-Cyclin B1, and anti-phospho-Aurora A (Thr 288) antibodies (see Table S3 in Supplementary Material), blocking and incubations were performed in TBS-Tween with 5% BSA or non-fat dry milk. Detection was performed using HRP-conjugated secondary antibodies (see Table S3 in Supplementary Material), with SuperSignal West Femto (Thermo Fisher Scientific) substrates. Membranes were imaged on a ChemiDoc MP system (Bio-Rad).

Aurora B inhibitor analysis was done as described above with the following adaptations: seeded HeLa cells were synchronized using a 2.5 mM double-thymidine block. Eight hours after release, cells were treated with 10 μM MG132 and DMSO or compound in dose–response for 3 h. After PBS washing, treated cells were harvested with sample buffer, and the total cell lysate was heated for 5 min at 95°C before sonication. Primary anti-phospho Histone H3(Ser 10), anti-phospho-Histone H3 (Ser 28), anti-Histone H3, anti-phospho-Aurora A(Thr 288)/Aurora B(Thr 232)/Aurora C(Thr 198), anti-Aurora B, and anti-Cyclin B1 antibodies were incubated and detected as described above. Primary and secondary antibody dilutions can be found in Table S3 in Supplementary Material.

### Aurora B Transcript Variant Analysis

HeLa total cellular RNA was prepared using RNeasy (Qiagen) according to the manufacturer’s instructions. Random primer-based cDNA synthesis was performed with MultiScribe reverse transcriptase (Applied Biosystems) from 500 ng RNA (20 μL reaction volume, 10 min at 25°C, 120 min at 37°C, 5 min at 85°C). The cDNA was diluted 1:5, and 10 μl was used in a 50 μl PCR reaction with Q5 DNA polymerase (New England Biolabs) and the following primers: GGTCATTTGTAGCCACATCCTGTC (specific to human Aurora B transcript 5; nucleotides 108–131 of RefSeq NM_001313951) and GCATCTGCCAACTCCTCCATGATC (universal primer for human Aurora B transcripts; nucleotides 687–664 of RefSeq NM_001313951). The PCR amplification conditions were (10 s at 98°C, 30 s at 69°C, 30 s at 72°C, 35 cycles). Reaction products were visualized by fluorescence on a 3% NuSieve GTG agarose gel. Identical PCR conditions were used for amplification with T7 and SP6 promoter sequences appended to the primers for direct sequencing after gel purification.

### Crystal Structure of Aurora A Bound to MK-5108

The kinase domain of human Aurora A (amino acids 123-390) was cloned into pET28a with an N-terminal 6XHis tag and an intervening rhinovirus 3C protease cleavage site. The protein was expressed in *E. coli* BL21 Rosetta 2(DE3) cells (Novagen) at 16°C overnight. Cells were harvested by centrifugation, resuspended in a buffer containing 50 mM Tris (pH 8.0), 300 mM NaCl, 40 mM imidazole, 20 mM MgCl_2_, 10% glycerol, 0.5 mM TCEP, and an EDTA-free protease inhibitor cocktail, and lysed using a microfluidizer. After clarification via centrifugation, the lysate was loaded onto a HisTrap HP column (GE Healthcare), and the bound protein was eluted in a buffer containing 50 mM Tris (pH 8.0), 300 mM NaCl, 200 mM imidazole, 20 mM MgCl_2_, 10% glycerol, and 0.5 mM TCEP. The tag was cleaved with Turbo3C protease (ETON) overnight at 4°C while being dialyzed against a buffer containing 20 mM Tris (pH 7.0), 200 mM NaCl, 20 mM MgCl_2_, 10% glycerol, and 0.5 mM TCEP. Since both the 6× His-tagged and untagged species bind metal affinity resins in this buffer, the cleavage reaction was loaded onto a HisTrap HP column and the untagged protein was selectively eluted in a buffer containing 20 mM Tris (pH 7.0), 200 mM NaCl, 40 mM imidazole, 20 mM MgCl_2_, 10% glycerol, and 0.5 mM TCEP. Trace amounts of the Turbo3C protease were removed using a GSTrap HP column (GE Healthcare). The untagged protein was further purified using size exclusion chromatography on a Superdex 75 16/600 column (GE Healthcare). The final eluate [in 20 mM Tris (pH 7.0), 200 mM NaCl, 20 mM MgCl_2_, 10% glycerol, and 0.5 mM TCEP] was concentrated to 6.2 mg/mL using Amicon Ultra 10K MWCO concentrators (Millipore), and MK-5108 was added from a 50 mM DMSO stock to a final concentration of 500 μM.

The inhibitor bound protein was crystallized by hanging drop vapor diffusion using a reservoir buffer consisting of 100 mM BisTris (pH 6.5), 30% PEG3350 at 21°C. A total of 1.5 μL protein solution was mixed with 1.5 μL reservoir buffer and sealed in a chamber containing 400 μL of reservoir solution. After 1 week, a rod-shaped crystal (~100 μm × 5 μm × 5 μm) was transferred to a cryoprotectant containing 100 mM BisTris (pH 6.5), 200 mM NaCl, 20 mM MgCl_2_, 25% PEG3350, 10% glycerol, 30 μM MK-5108, and flash-frozen in liquid nitrogen.

X-ray diffraction data were measured using Beamline 7-1 at the Stanford Synchrotron Radiation Lightsource and processed with HKL2000 (115). The structure was determined by molecular replacement using PHASER (116) and sequential searches with the large and then the small lobes of an ensemble model (PDB: 1MQ4, 2J4Z, 3FDN, 3LAU, 4UYN). Refinement was performed using PHENIX (117) interspersed with iterative cycles of rebuilding using Moloc (118). Figures were made using PyMol (Schrödinger).

## Author Contributions

JH, MY, and HL performed biochemical experiments to determine inhibitor potencies and selectivities. CdG, JA, YW, and RD performed cell-based experiments to determine inhibitor potencies and selectivities. CdG and DJ assessed inhibitor effects on cellular proliferation and apoptosis. DJ performed Aurora B and C expression analysis. MM and RD performed PCR analysis of Aurora B transcript variants. AM and AS determined the structure of Aurora A bound to MK-5108. TG supplied the inhibitors and arranged for kinome profiling. CdG, YW, AD, TG, and AS conceived and designed experiments. AD and AS wrote the manuscript with input from CdG and all other authors.

## Conflict of Interest Statement

The authors declare that the research was conducted in the absence of any commercial or financial relationships that could be construed as a potential conflict of interest.

## Supplementary Material

Movie S1 **Timelapse imaging of HeLa cells stably expressing GFP-PCNA; H2B-RFP**. Images were acquired at 12-min intervals; playback rate is 10 frames per second (7200 × real time).
