# Supplementary material for: A Cell Biologist’s Field Guide to Aurora Kinase Inhibitors
Source: Front Oncol. 2015 Dec 21;5:285. doi: 10.3389/fonc.2015.00285 (PMC4685510; doi:10.3389/fonc.2015.00285)
Supplement: Supplementary file 2 [file Table_1.PDF]

Table S1. Suppliers of Aurora inhibitors

Compound	Cas Registry Number	Vendors (Catalog Number)
VX-680 (Tozasertib)	639089-54-6	**Selleckchem (S1048)**, Apex Bio (A4111), LC Laboratories (T-2304)
MLN8054	869363-13-3	**Selleckchem (S1100)**, Apex Bio (A4114), MedKoo Bioscience (201930)
MLN8237 (Alisertib)	869363-13-3	**Selleckchem (S1133)**, MedKoo Bioscience (201931), Apex Bio (A4110)
MK-5108 (VX-689)	1010085-13-8	**Selleckchem (S2770)**, Apex Bio (A4120), MedKoo Biosciences (201917)
MK-8745	885325-71-3	**Selleckchem (S7065)**, Apex Bio (A8807), MedKoo Biosciences (406251)
Genentech Aurora A Inhibitor 1	1158838-45-9	**Selleckchem (S1451)**, Tocris Bioscience (5286), Apex Bio (A4126),
ZM447439	331771-20-1	**Selleckchem (S1103)**, Tocris Bioscence (2458), Apex Bio (A4113)
AZD1152-HQPA	722544-51-6	**Selleckchem (S1147) (*Incorrectly listed as Barasertib*)**, Active Biochem (A-1377), MedKoo Bioscience (200421)
Hesperadin (Hesperadin Hydrochloride)	422513-13-1	**Tocris Bioscience (3988)**, Selleckchem (S1529), Apex Bio (N1809)
GSK1070916	942918-07-2	**Selleckchem (S2740)**, MedKoo Biosciences (205477), Apex Bio (A4127)

BOLD = SUPPLIERS USED IN THIS STUDY
