# Supplementary material for: A Cell Biologist’s Field Guide to Aurora Kinase Inhibitors
Source: Front Oncol. 2015 Dec 21;5:285. doi: 10.3389/fonc.2015.00285 (PMC4685510; doi:10.3389/fonc.2015.00285)
Supplement: Supplementary file 3 [file Table_2.PDF]

**Table S2. Comparison of $K_i/IC_{50}$ values measured in this study to values reported in the literature**

## Aurora A:

| | $K_i$ (this study) | $K_i$ (Published) | Reference (PMID) | $IC_{50}$ (Published) | Reference (PMID) |
|---|---|---|---|---|---|
| **VX-680** | 1.03 nM | 1.4 nM<br><br>0.6 nM | 17705509 (ref. 70)<br>19447622 (ref. 42) | 36 nM | 18032922 (ref. 119) |
| **MK-5108** | <0.01 nM | 0.0155 nM | 24403173 (ref. 72) | 0.064 nM | 20053775 (ref. 56) |
| **MK-8745** | 0.06 nM | | | 0.6 nM | 22293494 (ref. 57) |
| **MLN8054** | 0.15 nM | 0.27 nM<br><br>7 nM | 20067443 (ref. 71)<br>26101564 (ref. 49) | 4 nM<br><br>1.0 nM | 17360485 (ref. 48)<br>19402633 (ref. 59) |
| **MLN8237** | 0.04 nM | 0.3 nM<br><br>0.136 nM | 26101564 (ref. 49)<br>24403173 (ref. 72) | 1.2 nM | 22016509 (ref. 55) |
| **Genentech Aurora Inh 1** | 0.57 nM | | | 3.4 nM | 19402633 (ref. 59) |
| **ZM447439** | 55.5 nM | | | 0.11 µM<br><br>1 µM<br><br>1 µM | 12719470 (ref. 44)<br>16912073 (ref. 120)<br>18032922 (ref. 119) |
| **AZD1152-HQPA** | 83.8 nM | <1 nM<br><br>0.36 nM | 17373783 (ref. 46)<br>17575233 (ref. 47) | | |
| **Hesperadin** | 1.21 nM | | | | |
| **GSK1070916** | 16.1 nM | | | | |

## Aurora A-TPX2:

| | $K_i$ (this study) | $K_i$ (Published) | Reference (PMID) | $IC_{50}$ (Published) | Reference (PMID) |
|---|---|---|---|---|---|
| **VX-680** | 4.55 nM | 5.9 nM | 17705509 (ref. 70) | 15 nM | 20624901 (ref. 121) |
| **MK-5108** | 0.04 nM | 2.17 nM | 24403173 (ref. 72) | | |

| | | | | | |
|---|---|---|---|---|---|
| **MK-8745** | 0.41 nM | | | | |
| **MLN8054** | 0.80 nM | 3.3 nM (AurA Ki = 0.75 nM) | 20067443 (ref. 71) | | |
| **MLN8237** | 0.23 nM | 1.86 nM | 24403173 (ref. 72) | | |
| **Genentech Aurora Inh 1** | 0.24 nM | | | 0.19X AurA | 19402633 (ref. 59) |
| **ZM447439** | 336.8 nM | | | 360 nM | 20624901 (ref. 121) |
| **AZD1152-HQPA** | 351.9 nM | | | | |
| **Hesperadin** | 1.37 nM | | | 11 nM | 20624901 (ref. 121) |
| **GSK1070916** | 130.2 nM | 492 nM | 19284385 (ref. 61) | | |

## Aurora B:

| | **$K_i$ (this study)** | **$K_i$ (Published)** | **Reference (PMID)** | **$IC_{50}$ (Published)** | **Reference (PMID)** |
|---|---|---|---|---|---|
| **VX-680** | 1.11 nM | 1.8 nM | 19447622 (ref. 42) | 18 nM<br>31 nM | 18032922 (ref. 119)<br>20624901 (ref. 121) |
| **MK-5108** | 1.49 nM | | | 14.1 nM | 20053775 (ref. 56) |
| **MK-8745** | 66.8 nM | | | 280 nM | 22293494 (ref. 57) |
| **MLN8054** | 1.65 nM | (6X AurA) | 20067443 (ref. 71) | 172 nM<br>9.3 nM | 17360485 (ref. 48)<br>19402633 (ref. 59) |
| **MLN8237** | 1.10 nM | | | 396.5 nM | 22016509 (ref. 55) |
| **Genentech Aurora Inh 1** | 156.2 nM | | | 3.4 µM | 19402633 (ref. 59) |
| **ZM447439** | 1.83 nM | | | 51 nM<br>0.13 µM<br>50 nM<br>50 nM | 20624901 (ref. 121)<br>12719470 (ref. 44)<br>16912073 (ref. 120)<br>18032922 (ref. 119) |

| | | | | | |
|---|---|---|---|---|---|
| **AZD1152-HQPA** | 0.02 nM | <1 nM<br><br>0.36 nM | 17373783 (ref. 46)<br>17575233 (ref. 47) | | |
| **Hesperadin** | 0.03 nM | | | 3 nM<br><br>250 nM | 20624901 (ref. 121)<br>12707311 (ref. 45) |
| **GSK1070916** | | 0.38 nM | 19284385 (ref. 61) | | |

## SUPPLEMENTAL REFERENCES


119. Tyler, R. K., Shpiro, N., Marquez, R., and Eyers, P. A. (2007) VX-680 inhibits Aurora A and Aurora B kinase activity in human cells. *Cell Cycle* **6**, 2846-2854

120. Girdler, F., Gascoigne, K. E., Eyers, P. A., Hartmuth, S., Crafter, C., Foote, K. M., Keen, N. J., and Taylor, S. S. (2006) Validating Aurora B as an anti-cancer drug target. *J Cell Sci* **119**, 3664-3675

121. Santaguida, S., Tighe, A., D'Alise, A. M., Taylor, S. S., and Musacchio, A. (2010) Dissecting the role of MPS1 in chromosome biorientation and the spindle checkpoint through the small molecule inhibitor reversine. *J Cell Biol* **190**, 73-87
