# Supplementary material for: A Cell Biologist’s Field Guide to Aurora Kinase Inhibitors
Source: Front Oncol. 2015 Dec 21;5:285. doi: 10.3389/fonc.2015.00285 (PMC4685510; doi:10.3389/fonc.2015.00285)
Supplement: Supplementary file 4 [file Table_3.PDF]

Table S3. Antibodies used in this study and supplier information

	Assay	Dilution/ Concentration	Vendor	Catalog #
**Primary Antibodies**				
mouse monoclonal anti-phospho-Lats2(Ser83) IgG2b	IF	1:1000	Cyclex	CY-M1020
mouse monoclonal anti-phospho-Ser/Thr-Pro MPM2 IgG1	IF	1:1000	Millipore	05-368MG
rat monoclonal anti-phospho-Histone H3(Ser28)	IF/WB	1:1000	Sigma	H 9908
mouse monoclonal anti-Histone H3	WB	1:1000	Cell Signaling	3638
mouse monoclonal anti-phospho-Histone H3(Ser10)	WB	1:1000	Millipore	05-1336
rabbit monoclonal anti-Aur A	WB	1:1000	Cell Signaling	4718
rabbit monoclonal anti-Aur B (N-terminal epitope)	WB	1:50000	Abcam	ab45145
rabbit polyclonal anti-Aur B (C-terminal epitope)	WB	1:5000	Abcam	ab70238
rabbit polyclonal anti-Cyclin B1	WB	1:1000	Cell Signaling	4138
rabbit monoclonal anti-phospho-Aur A(Thr288)	WB	1:1000	Cell Signaling	3079
rabbit monoclonal anti-phospho-Aur A(Thr288)/Aur B(Thr232)/Aur C(Thr198)	WB	1:2000	Cell Signaling	2914
**Secondary Antibodies**				
Alexa Fluor488-conjugated goat anti-mouse IgG2b	IF	1:1000	Life Technologies	A-21141
Alexa Fluor647-conjugated goat anti-mouse IgG1	IF	1:1000	Life Technologies	A10538
Cy3-conjugated goat F(ab')2-fragment anti-rat	IF	1 ug/ml	Jackson IR	112-166-072
HRP-linked donkey anti-rabbit	WB	1:20000	GE Healthcare	NA9340V
HRP-linked goat anti-rat	WB	1:20000	Cell Signaling	7077
HRP-linked sheep anti-mouse	WB	1:20000	GE Healthcare	NA9310V
