# Supplementary material for: A Cell Biologist’s Field Guide to Aurora Kinase Inhibitors
Source: Front Oncol. 2015 Dec 21;5:285. doi: 10.3389/fonc.2015.00285 (PMC4685510; doi:10.3389/fonc.2015.00285)
Supplement: Supplementary file 6 [file Table_5.PDF]

## Table S5: Data collection and refinement statistics

| **Data collection** | **MK-5108/ Aurora A Kinase Domain** |
|---|---|
| Resolution (Å) | 35.95 – 2.18 (2.22 – 2.18) |
| Space Group | $P2_12_12_1$ |
| Unit Cell Dimensions (a, b, c) Å | 51.85, 65.33, 77.27 |
| Unit cell Angles ($\alpha,\beta,\gamma$) ° | 90, 90, 90 |
| $I/\sigma$ (last shell) | 19.5 (1.9) |
| [1]$R_{sym}$ (last shell) | 0.090 (0.659) |
| [2]$R_{meas}$ (last shell) | 0.097 (0.756) |
| Completeness (last shell) % | 99.7 (97.1) |
| Number of reflections | 94967 |
| *unique* | 14199 |
| Multiplicity (last shell) | 6.7 (4.1) |
| | |
| **Refinement** | |
| Resolution (Å) | 33.25 – 2.18 (2.26-2.18) |
| No. of reflections | |
| *working* | 12737 |
| *free* | 1418 |
| [3]$R_{work}$ (last shell) (%) | 18.55 (25.96) |
| [3]$R_{free}$ (last shell) (%) | 23.74 (33.55) |
| | |
| **Structure/Stereochemistry** | |
| No. of atoms | |
| *protein* | 2091 |
| *solvent* | 74 |
| *ligand* | 31 |
| r.m.s.d. bond lengths (Å) | 0.006 |
| r.m.s.d. bond angles (°) | 1.108 |
| Average B-Factor | 49.34 |
| Protein Data Bank ID[4] | 5EW9 |

[1]$R_{sym} = \sum\sum_j|I_j - \langle I\rangle|/\sum I_j$, where $I_j$ is the intensity measurement for reflection j and $\langle I\rangle$ is the mean intensity for multiply recorded reflections.

[2]$R_{meas} = \sum_h [\ \sqrt{(n/(n\text{-}1))}\ \sum_j [I_{hj} - \langle I_h\rangle] / \sum_{hj}\langle I_h\rangle$, where $I_{hj}$ is a single intensity measurement for reflection h, $\langle I_h\rangle$ is the average intensity measurement for multiply recorded reflections, and $n$ is the number of observations of reflection h.

[3]$R_{work,\ free} = \sum||F_{obs}| - |F_{calc}||/|F_{obs}|$, where the working and free $R$-factors are calculated using the working and free reflection sets, respectively.

[4]Coordinates and structure factors have been deposited with the Protein Data Bank (http://www.pdb.org) with the noted accession code.
