# Supplementary material for: A Cell Biologist’s Field Guide to Aurora Kinase Inhibitors
Source: Front Oncol. 2015 Dec 21;5:285. doi: 10.3389/fonc.2015.00285 (PMC4685510; doi:10.3389/fonc.2015.00285)
Supplement: Supplementary file 7 [file Table_6.PDF]

Table S6. Aurora A residues interacting with MK-5108
(equivalent Aurora B residues in parentheses – differences bolded)

Leu 139	(Leu 83)
Gly 140	(Gly 84)
Ala 141	**(Lys 85)** (Main chain atom interactions only)
Gly 142	(Gly 86)
Val 147	(Val 91)
Ala 160	(Ala 104)
Lys 162	(Lys 106)
Leu 194	(Leu 138)
Leu 210	(Leu 154)
Glu 211	(Glu 155)
Tyr 212	(Tyr 156)
Ala 213	(Ala 157)
Pro 214	(Pro 158)
Gly 216	(Gly 160)
Thr 217	**(Glu 161)** (Side chain interactions)
Arg 220	**(Lys 164)** (Side chain interactions)
Glu 260	(Glu 204)
Asn 261	(Asn 205)
Leu 263	(Leu 207)
Ala 273	(Ala 217)
Phe 275	(Phe 219)
Trp 277	(Trp 221)
