# Supplementary material for: A Cell Biologist’s Field Guide to Aurora Kinase Inhibitors
Source: Front Oncol. 2015 Dec 21;5:285. doi: 10.3389/fonc.2015.00285 (PMC4685510; doi:10.3389/fonc.2015.00285)
Supplement: Supplementary file 8 [file Image_1.PDF]

A

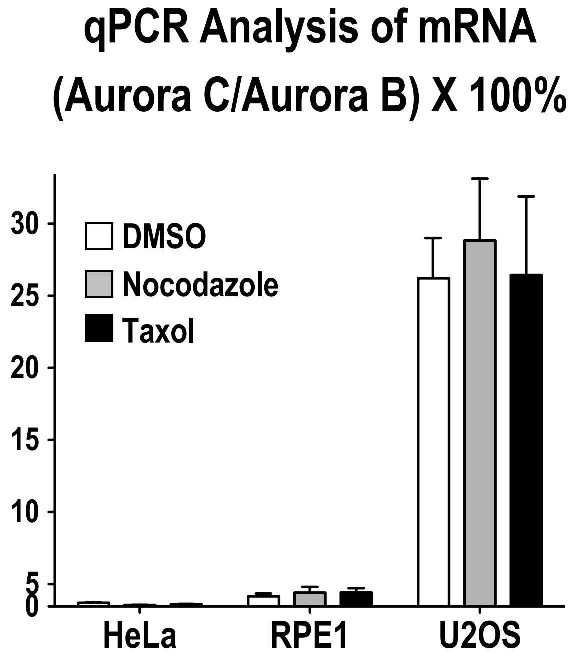

B

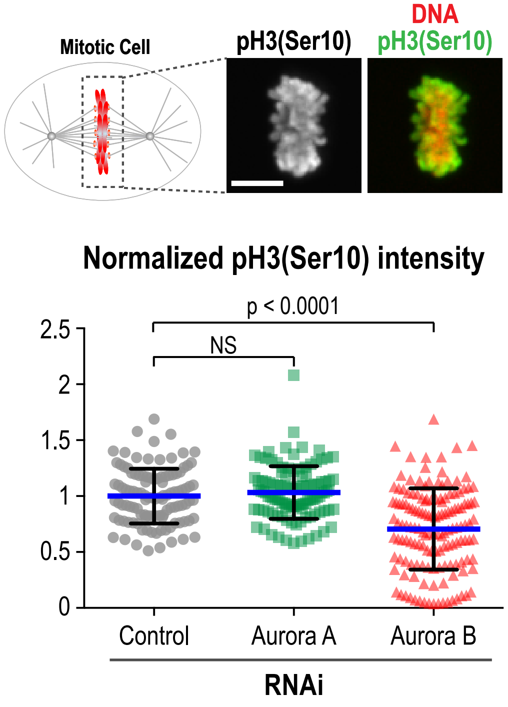

Figure S1. qPCR analysis of Aurora C mRNA levels and effect of Aurora A or B RNAi on pH3(Ser10) labeling.
(**A**) qPCR analysis of Aurora C versus Aurora B mRNA levels in the indicated cell lines. Total RNA was extracted using the Qiagen RNeasy Mini Kit (Qiagen). Total RNA was reverse-transcribed using the High Capacity cDNA Reverse Transcription Kit and qPCR was performed using Taqman Gene Expression Assays (Life Technologies). Aurora B mRNA levels are comparable between asynchronously growing HeLa, U2OS and RPE1 cells (Aurora B(U2OS)/Aurora B(HeLa)=120%; Aurora B(RPE1)/Aurora B(HeLa)=102%). Hence, the Aurora C/Aurora B mRNA ratios of U2OS and RPE1 cells are higher than that of HeLa cells predominantly due to greater levels of Aurora C mRNA.
(**B**) Effect of Aurora A versus Aurora B RNAi on pH3(Ser 10) labeling. Image shown is of a mitotic HeLa cell labeled for pH3(Ser 10) and DNA. p-values are from unpaired t-tests. Scale bar, 10 µm.
