# Supplementary material for: A Cell Biologist’s Field Guide to Aurora Kinase Inhibitors
Source: Front Oncol. 2015 Dec 21;5:285. doi: 10.3389/fonc.2015.00285 (PMC4685510; doi:10.3389/fonc.2015.00285)
Supplement: Supplementary file 9 [file Image_2.PDF]

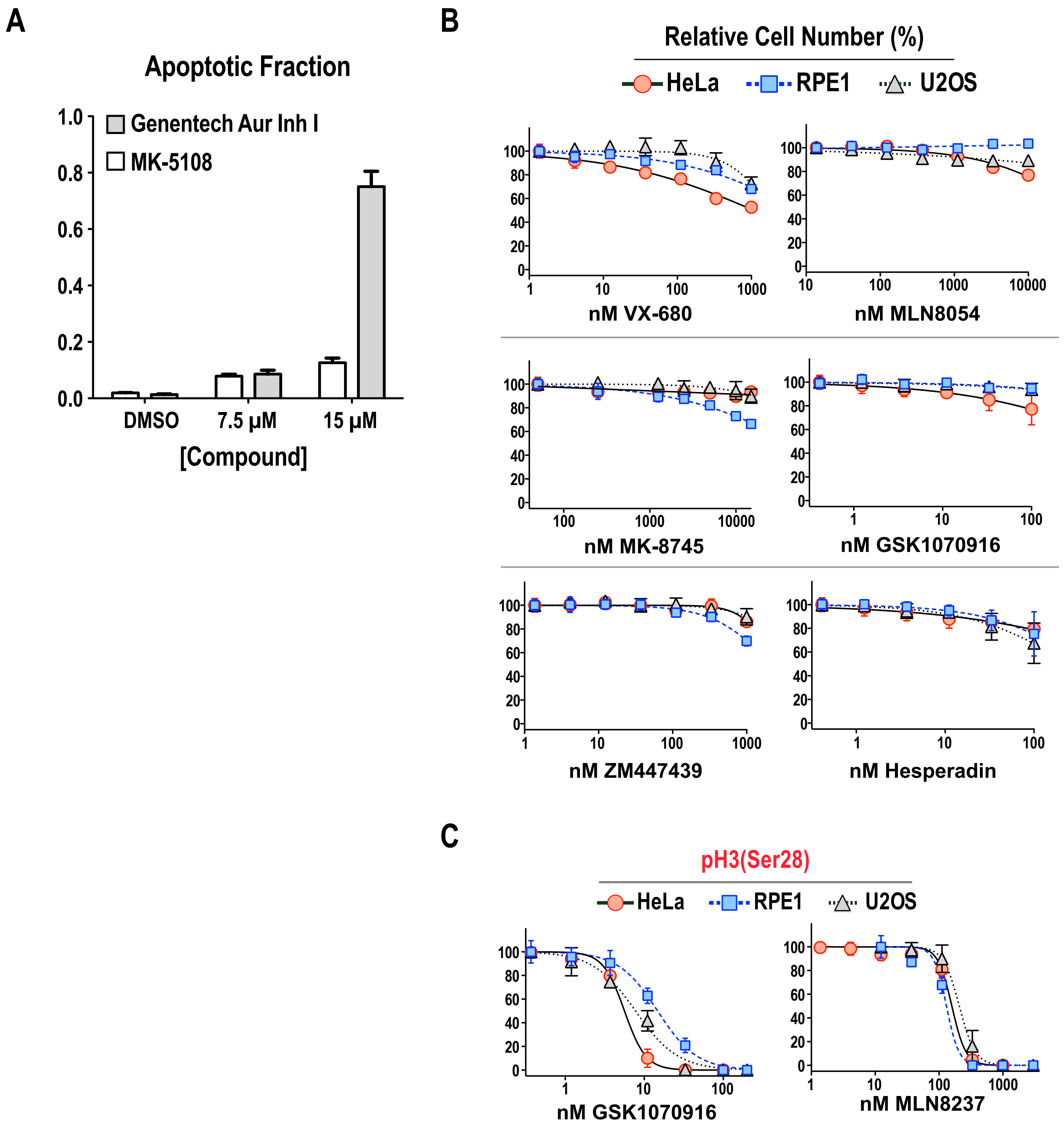

Figure S2. Measurement of apoptosis, cell proliferation, and pH3(Ser 28) labeling following inhibitor treatments.
(A) Fraction of apoptotic cells for the indicated conditions.
(B) Dose-response curves measuring cellular proliferation for the indicated conditions, performed as in Fig. 4D.
(C) Dose-response data for pH3(Ser 28) labeling intensity plotted for all 3 cell lines for the indicated inhibitors.
