# Supplementary material for: A Cell Biologist’s Field Guide to Aurora Kinase Inhibitors
Source: Front Oncol. 2015 Dec 21;5:285. doi: 10.3389/fonc.2015.00285 (PMC4685510; doi:10.3389/fonc.2015.00285)
Supplement: Supplementary file 10 [file Image_3.PDF]

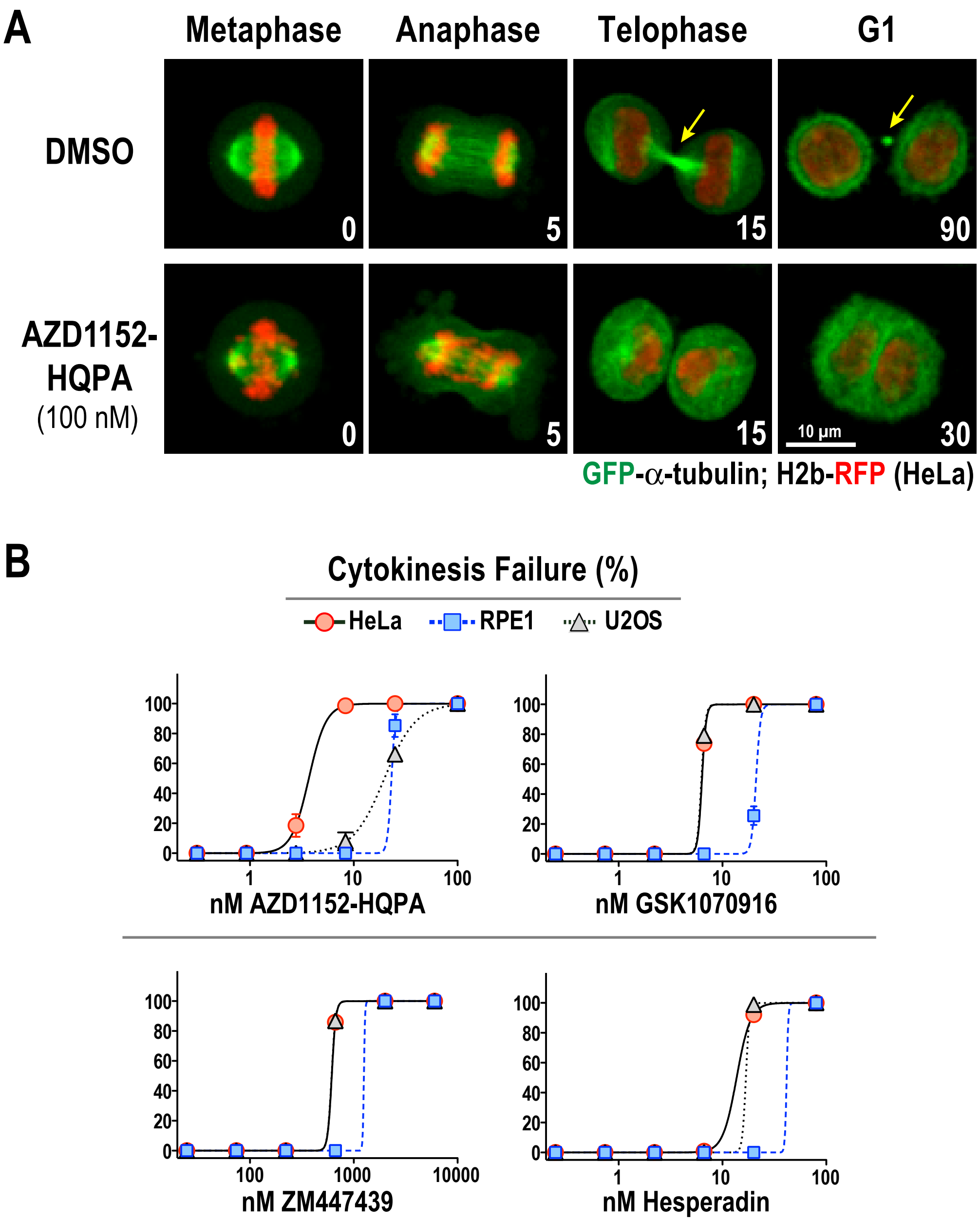

Figure S3. Dose-response analysis of cytokinesis failure with inhibitors specifically targeting Aurora B.
(**A**) Images from timelapse sequences showing a control DMSO-treated HeLa cell and a 100 nM AZD1152-HQPA-treated cell. The midbody is indicated in the control cell (yellow arrows).
(**B**) Dose response curves for cytokinesis failure in the three tested cell lines for the indicated 4 Aurora B-specific inhibitors. Each point represents the mean of 2 sets of measurements (average 75 cells per set). As expected, at concentrations of the 4 Aurora B inhibitors that eliminate pH3(Ser 28) labeling, no midbody assembly was observed and cytokinesis failed, resulting in binucleate cells. Notably, cytokinesis failure exhibited a sharp dose-response, suggesting the existence of a threshold of Aurora B activity, above which cytokinesis succeeds and below which it fails. This result also suggests the potential to titrate inhibitor dosage in order to analyze Aurora B functions that may require different thresholds of kinase activity. We note that Hesperadin, while as potent as AZD1152-HQPA and GSK1070916, appeared to be unstable under live imaging conditions, as its ability to block cytokinesis diminished significantly after 12 hours of imaging. This observation suggests that Hesperadin may not be optimal for long-term live imaging studies.
