# Supplementary material for: A Cell Biologist’s Field Guide to Aurora Kinase Inhibitors
Source: Front Oncol. 2015 Dec 21;5:285. doi: 10.3389/fonc.2015.00285 (PMC4685510; doi:10.3389/fonc.2015.00285)
Supplement: Supplementary file 11 [file Image_4.PDF]

de Groot et al. Figure S4

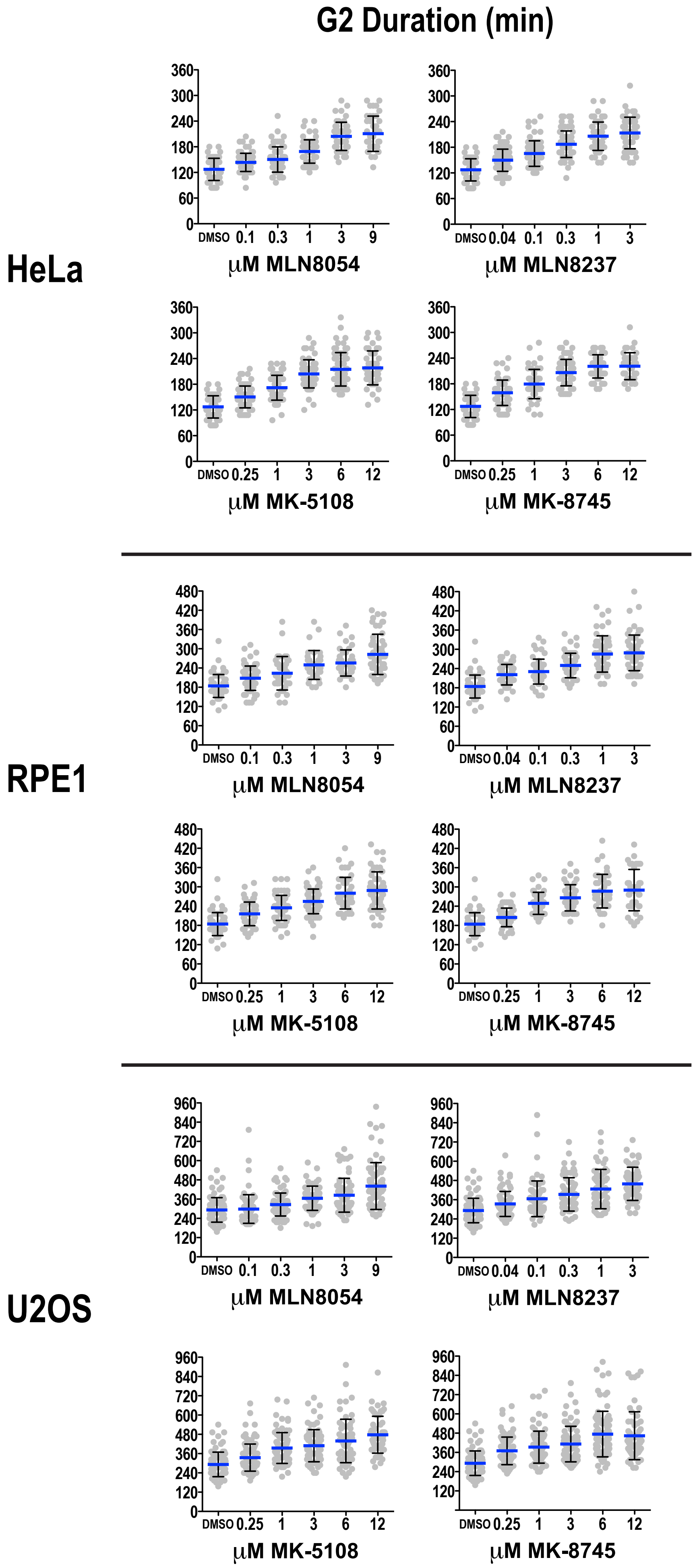

Figure S4. Raw data of G2 duration measurements. The mean values for the data shown here are plotted in Fig. 7.
