# Supplementary material for: A Cell Biologist’s Field Guide to Aurora Kinase Inhibitors
Source: Front Oncol. 2015 Dec 21;5:285. doi: 10.3389/fonc.2015.00285 (PMC4685510; doi:10.3389/fonc.2015.00285)
Supplement: Supplementary file 12 [file Image_5.PDF]

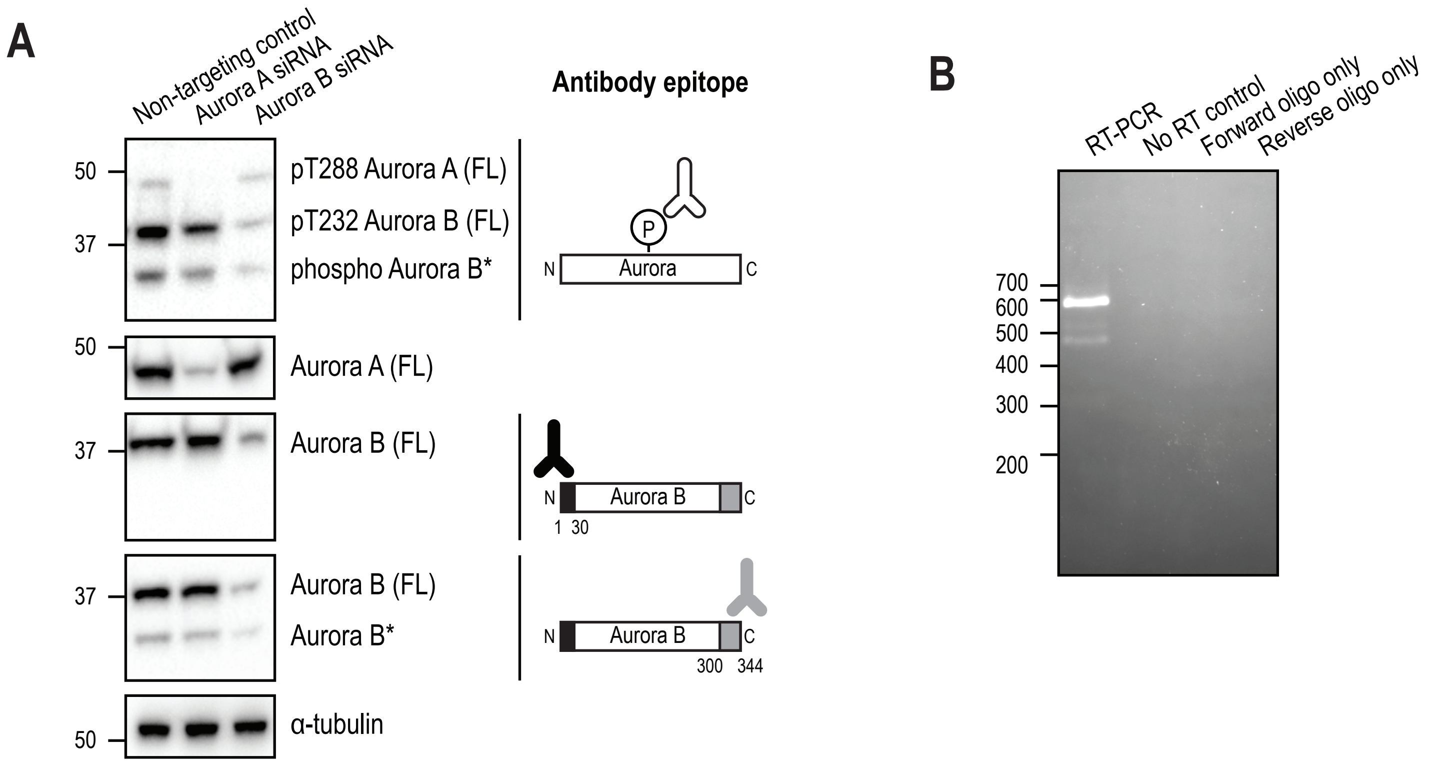

Figure S5. Validation of the pAuroraA(Thr 288)/pAuroraB(Thr 232)/pAuroraC(Thr 198) antibody.

(A) RNAi depletions were performed as described in the Methods section except that unmodified HeLa cells were used, and were replated after transfection into 6- or 12-well dishes. This monoclonal antibody (Cell Signaling; Table S3) recognizes three bands (~48 kD, ~39 kD, ~35 kD) in HeLa cell extracts (top panel, first lane). The ~48 kD band migrates near the expected position of full-length Aurora A (UniProt O14965; molecular weight = 45,809) and virtually disappears (top panel, middle lane) with treatment with an siRNA targeting Aurora A (second panel, middle lane) and Aurora A inhibitor treatment (Fig. 8A), indicating that this band represents full-length Aurora A phosphorylated on Thr 288. The ~39 kD band migrates near the expected position of full-length Aurora B (UniProt Q96GD4-1; Isoform 1; molecular weight = 39,311), and an equivalent band can be found at approximately the same position in blots using both 1) a monoclonal antibody (Abcam; Table S3) whose epitope is within residues 1-30 (N-terminus) of full-length Aurora B (third panel, first lane) and 2) a polyclonal antibody whose epitope is within residues 300-344 (C-terminus) of Aurora B (fourth panel, first lane) (Abcam; Table S3). Since it significantly diminishes in intensity with treatment with Aurora B siRNA in the phosphoAurora (top panel, third lane) and both total Aurora B antibody blots (third and fourth panels, third lane), the ~39 kD band in the phosphoAurora antibody blot (which is also sensitive to inhibitor treatment – Fig. 8B; Fig. S6) represents full length Aurora B phosphorylated on Thr 232. While the ~35 kD band migrates near the expected position of full-length Aurora C (UniProt Q9UQB9-1; Isoform 1; molecular weight = 35,591), an equivalent band is also evident in the blot with the antibody to the C-terminus of Aurora B (fourth panel, first lane). Since the intensity of this band is also sensitive to Aurora B siRNA treatment in both of these blots (first and fourth panel, third lane), we believe the ~35 kD band (Aurora B*) is an N-terminally truncated, alternatively spliced form of Aurora B, possibly Isoform 4 (see (B)). α-tubulin serves as a loading control for these immunoblots. FL=full-length.

(B) Using a set of primers specific for Aurora B transcript variant 5 (RefSeq NM_001313951), the expected 580 base pair band could be specifically amplified from HeLa total RNA after reverse transcription. This message encodes a 303 amino acid form of Aurora B (UniProt Q96GD4-4; Isoform 4; molecular weight = 34,760) that lacks the N-terminal 41 residues of the full-length protein. No PCR product was observed in the absence of reverse transcription or when only one of the two primers was used. Direct sequencing of the 580 base pair PCR product confirmed its identity. RT=reverse transcription
