# Supplementary material for: A Cell Biologist’s Field Guide to Aurora Kinase Inhibitors
Source: Front Oncol. 2015 Dec 21;5:285. doi: 10.3389/fonc.2015.00285 (PMC4685510; doi:10.3389/fonc.2015.00285)
Supplement: Supplementary file 13 [file Image_6.PDF]

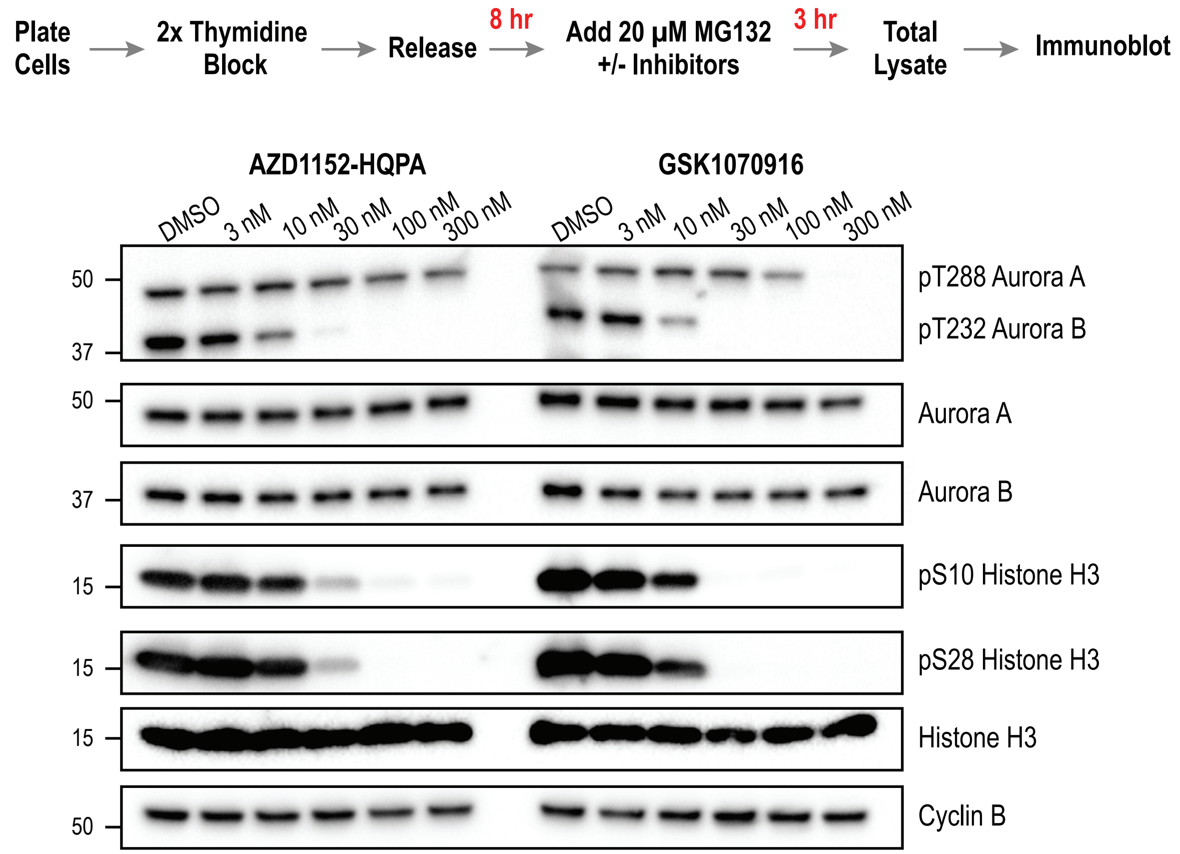

Figure S6. Immunoblotting of activation loop phosphorylation of Aurora A/B and of pH3(Ser 28) and pH3(Ser 10) in HeLa cells treated with AZD1152-HQPA and GSK1070916 at higher concentrations. In an independent experiment, mitotic lysates and blots were generated as in Fig. 8B except that a top dose of 300 nM was used.
