# Supplementary material for: A Cell Biologist’s Field Guide to Aurora Kinase Inhibitors
Source: Front Oncol. 2015 Dec 21;5:285. doi: 10.3389/fonc.2015.00285 (PMC4685510; doi:10.3389/fonc.2015.00285)
Supplement: Supplementary file 14 [file Image_7.PDF]

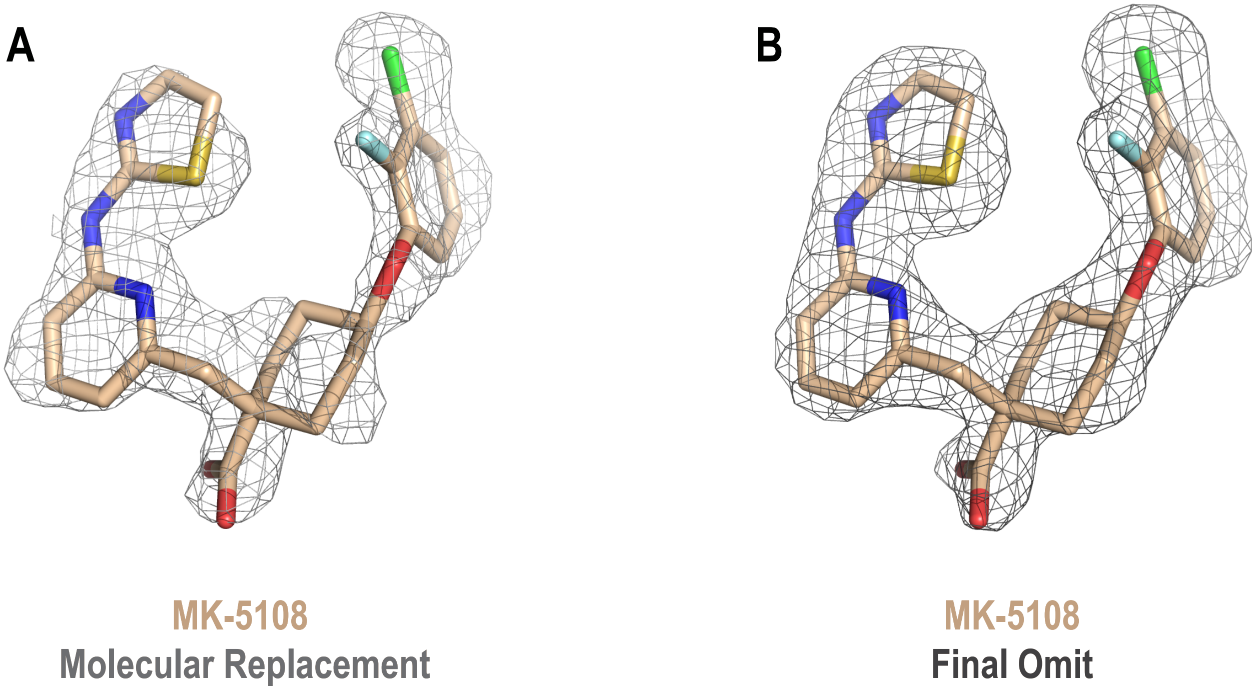

Figure S7. Electron density for MK-5108.
(A) Inhibitor region of the $2F_o$-F_c map calculated using the kinase domain model alone after molecular replacement and one round of refinement (contoured at 1σ). This density was used to unambiguously place the initial model of MK-5108.
(B) Inhibitor region of the $2F_o$-F_c map calculated with the coordinates of the final structure with the compound omitted (contoured at 1σ). The final model for MK-5108 is shown in both panels.
